# An Advanced Coarse-Grained Nucleosome Core Particle Model for Computer Simulations of Nucleosome-Nucleosome Interactions under Varying Ionic Conditions

**DOI:** 10.1371/journal.pone.0054228

**Published:** 2013-02-13

**Authors:** Yanping Fan, Nikolay Korolev, Alexander P. Lyubartsev, Lars Nordenskiöld

**Affiliations:** 1 Division of Structural Biology and Biochemistry, School of Biological Sciences, Nanyang Technological University, Singapore, Singapore; 2 Division of Physical Chemistry, Department of Materials and Environmental Chemistry, Arrhenius Laboratory, Stockholm University, Stockholm, Sweden; University of Akron, United States of America

## Abstract

In the eukaryotic cell nucleus, DNA exists as chromatin, a compact but dynamic complex with histone proteins. The first level of DNA organization is the linear array of nucleosome core particles (NCPs). The NCP is a well-defined complex of 147 bp DNA with an octamer of histones. Interactions between NCPs are of paramount importance for higher levels of chromatin compaction. The polyelectrolyte nature of the NCP implies that nucleosome-nucleosome interactions must exhibit a great influence from both the ionic environment as well as the positively charged and highly flexible N-terminal histone tails, protruding out from the NCP. The large size of the system precludes a modelling analysis of chromatin at an all-atom level and calls for coarse-grained approximations. Here, a model of the NCP that include the globular histone core and the flexible histone tails described by one particle per each amino acid and taking into account their net charge is proposed. DNA wrapped around the histone core was approximated at the level of two base pairs represented by one bead (bases and sugar) plus four beads of charged phosphate groups. Computer simulations, using a Langevin thermostat, in a dielectric continuum with explicit monovalent (K^+^), divalent (Mg^2+^) or trivalent (Co(NH_3_)_6_^3+^) cations were performed for systems with one or ten NCPs. Increase of the counterion charge results in a switch from repulsive NCP-NCP interaction in the presence of K^+^, to partial aggregation with Mg^2+^ and to strong mutual attraction of all 10 NCPs in the presence of CoHex^3+^. The new model reproduced experimental results and the structure of the NCP-NCP contacts is in agreement with available data. Cation screening, ion-ion correlations and tail bridging contribute to the NCP-NCP attraction and the new NCP model accounts for these interactions.

## Introduction

**Figure 1:**
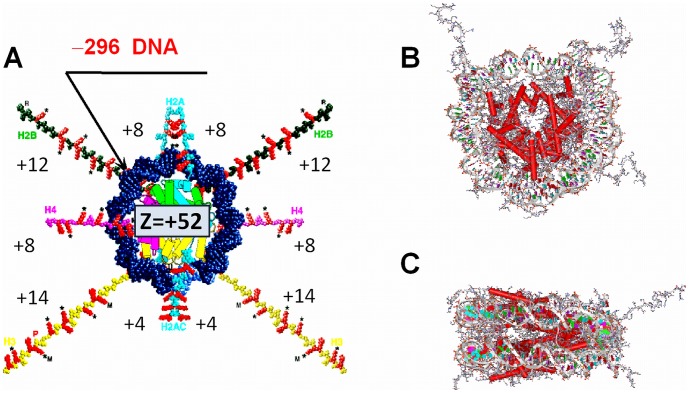
Structure of the nucleosome core particle. (**A**). Schematic representation of the NCP with histone tails stretched out. The net positive charge of the globular (core) part of the histone octamer (Z = +52e) and of the histone tails is shown. (**B**) The nucleosome crystal structure at 1.9 Å resolution (pdb 1KX5 [2]). (**C**) Side view of the NCP to highlight its wedge shape.

In the nucleus of all eukaryotic cells, the carrier of the genetic information, DNA is highly compacted by nuclear proteins into chromatin. The first level of DNA compaction comprises linear domains of DNA-histone complexes, the nucleosomes, which are highly uniform and conserved. The regular central part of the nucleosome is the nucleosome core particle (NCP), a wedge-shaped complex of 145–147 bp of DNA wrapped as a 1.75-turn superhelix around an octamer of histone proteins composed of two copies of the four histone H2A, H2B, H3 and H4 [1]–[3] (Fig. 1). Each of the core histones has an unstructured, flexible and positively charged N-terminal domain, the “histone tail” [1], [2], [4]. Double-stranded linker DNA of variable length connects the NCPs with each other to form a nucleosome array, which is complemented by linker histones and in vitro condenses fibre in a salt-, linker histone-, histone tail-dependent manner [5]. The higher levels of chromatin spatial organization are less known; it is believed that in vivo the chromatin compaction proceeds by the nucleosome array forming a 30 nm fibre [6]–[9] but this was recently questioned [10]–[12] (and references cited in [11]). It is clear that both in vitro and in vivo, the histone tails are necessary for secondary and tertiary condensation of chromatin [5], and that they participate in both intra- and inter-array nucleosome-nucleosome interactions [13]. The histone tails are important factors regulating replication, transcription and repair in the living cells [14]. The detailed physical and chemical mechanism of this regulation is still not fully understood. However, it is clear that the polyelectrolyte nature of DNA and the large positive charge of the histones, which neutralize about half of the DNA charge in the NCP (Fig. 1) means that the structural and dynamic properties of chromatin are governed by long-range electrostatic interactions with the mobile ions present in the system [15]. Rigorous theoretical approaches addressing these electrostatic effects are still lacking, though [15], [16].

Isolated NCPs display properties similar to chromatin fibres in terms of the experimentally observed salt-induced self-association [17]–[21]. The work of Livolant and colleagues analysed the phase behaviour of NCP solutions and gave information on the structural details of the NCP-NCP interactions [17]–[25]. It has also been shown that cation-induced NCP and nucleosome array aggregation displays a behaviour similar to other polyelectrolyte systems such as DNA [15], [26]–[28]. Aggregation of NCPs is dependent on the charge and nature of cations present in solution [17], [20], [26], [28], [29] and it was concluded that the histone tails mediate NCP-NCP contacts in a salt-dependent manner [18], [19], [21], [25], [28]. A compulsory feature of condensed nucleosome and chromatin structures is the formation of NCP stacking; close NCP-NCP contacts between the relatively flat surfaces of the histone octamer core on both sides of the cylindrical wedge-shaped NCP. Such close stacking has been observed in NCP crystals [1]–[3], [30], [31], in NCP liquid crystalline phases [20], [22]–[24], [32], in the crystal of the tetranucleosome [33], in folded nucleosome arrays [6], [34] and in cryo-microscopy images of frozen isolated native chromatin [35].

The polyelectrolyte nature of DNA and the high positive charge of the histones would strongly suggest that the contribution of electrostatic interactions to the stability, structure and dynamics of NCP-NCP interaction is profound and that nucleosome-nucleosome interactions should display a pronounced sensitivity to the ionic environment. Indeed, single molecule force spectroscopy measurements of chromatin fibre stretching gave an estimate for the energy of the NCP-NCP stacking at about 35 kJ/mol [9] at “physiological” salt (100 mM K^+^, 2 mM Mg^2+^) whereas experiments at lower salt concentration (40 mM Na^+^) reported a value about four times lower [36]. The dynamic nature of the mobile cations, the flexible unstructured histone tails, the highly complex and variable structure of chromatin significantly restricts application of single crystal X-ray crystallography and other structural methods in the investigation of chromatin folding and dynamics. After decades of efforts, only recently there were a few structures beyond the NCP reported; including the tetranucleosome [33], complexes of the NCP with the RCC1 chromatin factor [37], the chromatin remodelling factor ISW1a [38] and the silencing Sir3 BAH domain [39].

In all structures of the NCP and its complexes, the location and structure of the histone tails are not fully resolved. Consequently, computer modelling approaches can contribute substantially to understanding chromatin organization and dynamics where NCP-NCP contacts play an important role and are mediated by histone tails. Fully atomistic explicitly and implicitly solvated NCPs were studied in a number of works [40]–[46], but the simulation time was too short to describe and understand conformational changes. Much larger simulation setups are needed to model NCP-NCP interactions and chromatin folding; the size of these simulations and time length required presently prohibits such modelling at the atomistic level. Therefore, coarse-grained modelling of such large systems becomes necessary.

A number of coarse-grained models of DNA [47]–[51] and chromatin [43], [52]–[68] have been developed recently (reviewed in [16]). Most of the chromatin models aim at describing and understanding the nature of the folded 30 nm fibre chromatin structure. The histone tails are omitted in most of the models and the nucleosome-nucleosome interactions are modelled with implicit effective attractive potentials. Moreover, the physical origin of salt-dependent chromatin folding is usually not considered in these models. Many studies did not include explicit description of counterions, but instead used Debye-Hückel approximations [56], [59], [62], [63] for the electrostatic interactions or effective nucleosome-nucleosome potentials [55], [58], [60], [61]. As a result, entropy effects due to release of monovalent cations, ion-ion correlations and ion competition were not explicitly accounted for.

An accurate account of electrostatic forces in a system of strongly charged macroions necessitates explicit representation of mobile ions in the simulations. We have previously performed modelling of NCP and chromatin fibre solutions using very simple coarse-grained models of the NCP [53], [64] and the nucleosome array [69], [70]. The major advantage of our simplified modelling was that the long-range electrostatic interactions were described by assigning physically adequate charges to the NCP array and by explicit inclusion of mobile ions that emphasizes the polyelectrolyte nature of the system. Explicit modelling of flexible histone tails was another advantageous feature. It was found that this approach satisfactorily reproduced the experimentally observed salt-dependent NCP aggregation [53], [64] and chromatin folding [69] in the presence of Mg^2+^, cobalt(III)hexammine (Co(NH_3_)_6_^3+^, CoHex^3+^), spermidine^3+^ and spermine^4+^ cations. Despite an approximate coarse-graining of the NCP, the electrostatic approach did well describe the non-specific long-range electrostatic interaction that is the determining factor behind salt-induced folding observed in chromatin.

In order to understand and model the forces and molecular interactions responsible for the detailed structure of the higher order chromatin folding, its compaction and regulation by the ionic environment and by the chemical modifications on the histone tails, the model previously used must be improved. Such an improvement should include accurate representation of the NCP geometry, an adequate description of the excluded volume effects of the histone tails and ions, and the specific assignment of charges on the surface of the DNA, the histone tails and on the surface of the globular part of the histone octamer.

In this paper we present and validate the design of a new coarse-grained model of the NCP which satisfies these requirements. This new NCP model takes place between the atomistic level of descriptions and the simple polyelectrolyte models previously used by our group. Several simulation have been performed using this model for a single NCP as well as for ten NCPs in the presence of monovalent (K^+^), divalent (Mg^2+^) and trivalent (CoHex^3+^) counterions. Aggregation of NCPs induced by these cations has been studied experimentally [20], [28], [29] and the formation of NCP-NCP ordered columnar phases was reported upon addition of Mg^2+^ and polyamines [20]. The present simulations revealed that Mg^2+^ and CoHex^3+^ ions can promote NCP-NCP aggregation with NCP-NCP stacking contacts remarkably similar to the ones observed in condensed phases of the nucleosome core particles [20], [22], [24].

## Methods

### Principles of the Advanced Model

**Figure 2:**
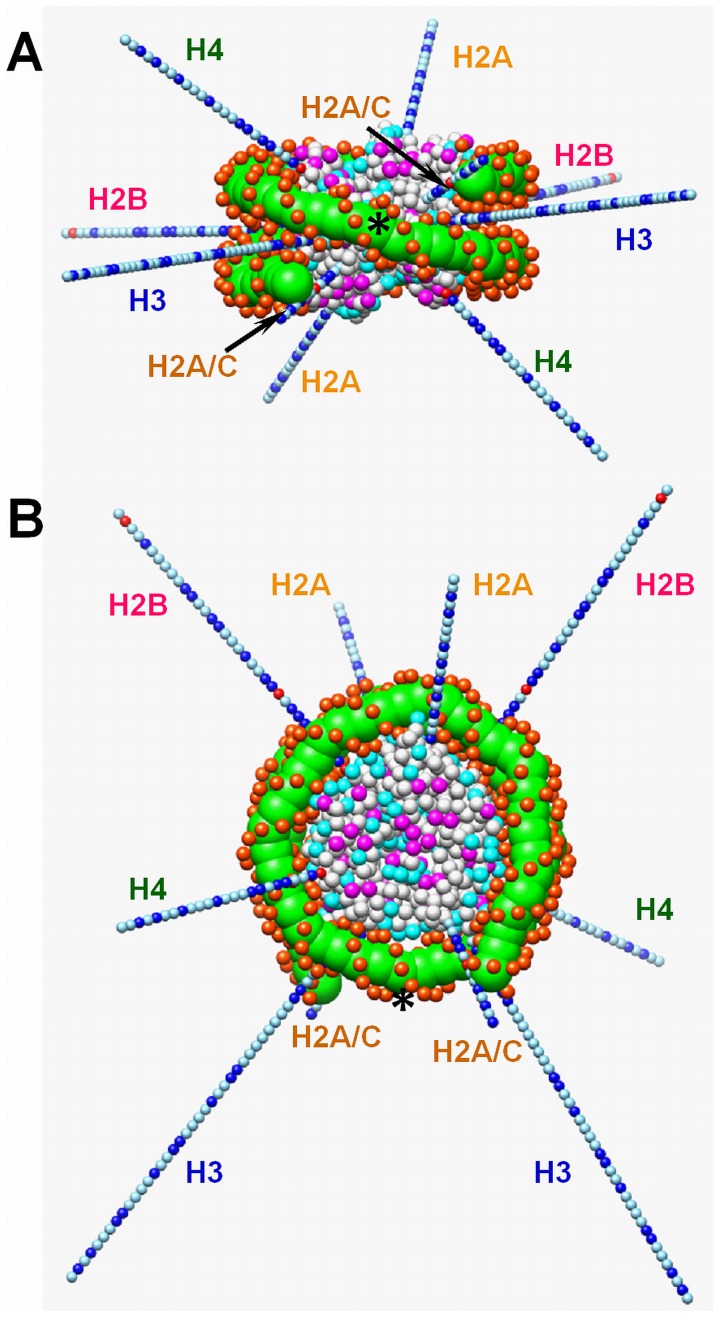
Coarse-grained model of the NCP. Side (**A**) and top (**B**) views of the model. Grey spheres represent space-filling approximation of the histone octamer core with the negatively and positively charged amino acid highlighted in magenta and cyan respectively; green and orange-red spheres represent the DNA structure where orange-red particles mimic the distribution of the negative phosphate groups. The histone tails are shown as light blue (neutral), blue (positive) and red (negative) particles, each representing one amino acid. Histone tails are labelled with the colour code taken from the first crystal structure of the NCP [1]: yellow (H2A, N- and C-termini), pink (H2B), blue (H3), green (H4). Black stars show the positions of the dyad axis.

The purpose of developing a novel coarse-grained NCP model is to reproduce in realistic details the shape of NCP including the wedge-shaped histone octamer core, and to provide an accurate description of the electrostatic interactions, which includes mobile ions, charges of DNA phosphate groups as well as charges of the histone tails and in the core region. To satisfy this level of precision, we have chosen to describe the system via a collection of coarse-grained beads representing different fragments of the histone octamer and DNA, which interact with other beads by a sum of Coulombic and short-range repulsive Lennard-Jones interactions. The beads belonging to the DNA and the histone octamer are held together in a single NCP by a network of harmonic bond and angle interactions which are described below. The NCP model is displayed in Fig. 2.

The mobile ions are also represented by separate beads. The solvent is treated implicitly by a continuum dielectric model. Thus, the total interaction potential (potential energy) of the system is:
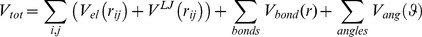
(1)where (i,j) are bead numbers.

The Coulombic electrostatic potential for charges (*q_i_, q_j_*) in a dielectric continuum with permittivity ε = 78 is defined in a standard way:
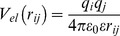
(2)


The short-range Lennard-Jones potential is set repulsive and calculated by the equation:
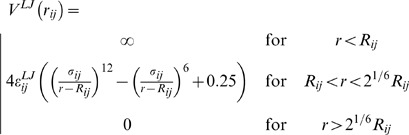
(3)where the hard core particle contact distance *R_ij_* = *R_i_*+*R_j_* is determined by the values of the hard core radii of both beads. The parameters σ*_ij_* and 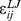 were set to σ*_ij_* = 4 Å and ε*_ij_* = 1 (*k_B_T* units) for all interactions. Thus, the effective radius of each particle was determined by a sum of the soft radius of σ_ij_/2 = 2 Å and the hard radius *R_i_* which was defined separately for each particle type.

In our model, we consider the solvent as a dielectric medium. The interaction potentials thus represent an approximation to the solvent-mediated effective potential between the charged particles in the solvent medium. Thus, ε is a parameter of the effective ion-ion potential. Available studies at an all atom level have shown that the effective (mean force) potentials of ions in water usually have one or two oscillations around the Coulomb potential with ε = 78 at small (within 8 Å) distances between the ions [71]). These oscillations reflect the molecular nature of the solvent. However, for many applications, including strong polyelectrolytes and high salt concentrations, continuum dielectric models with constant dielectric permittivity have been very successful [72], [73].

In a dielectric continuum model the ion interactions and competitions (especially multivalent ones) play a large role. It is important to assign appropriate radii, reflecting the effective hydration of the ions. Here, the following values of the hard radii were used: R(K^+^) = 0, R(Mg^2+^) = 0.5 Å and R(CoHex^3+^) = 1.5 Å, which corresponds to effective radii of 2, 2.5 and 3.5 Å for mono, di- and three-valent ions, respectively. The phosphate group of DNA was approximated as a bead with effective radius 3.0 Å. The choice of sizes for K^+^, Mg^2+^ and Co(NH_3_)_6_^3+^ is justified by our previous extensive comparison of experimental as well as computer modelling results of ion-DNA, ion-NCP and ion-chromatin interactions and ion-ion competitions [15], [16], [74]–[81]. We compared experimental and computationally modelled ion-DNA binding and competition effects for mixtures of monovalent cations K^+^, Na^+^, Li^+^ [75]; divalent and monovalent ions, K^+^, Na^+^, Ca^2+^, Mg^2+^ [76]; and for the trivalent and monovalent cations, K^+^, Na^+^, CoHex^3+^ [77]. In these studies the dielectric continuum approximation for the solvent was used and effective radii of the charged particles and resolution of the coarse-grained model of DNA (cylinder with attached phosphate groups) were similar to the sizes adopted in this work. These values are typical for hydrated ion radii used in a number of models and well reproduce thermodynamic properties and the features of ion distributions compared to all-atom simulations [74], [76], [77], [82], [83].

The bond and angle potentials for bound sites were calculate by the equations:
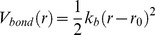
(4)

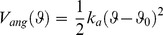
(5)where *k_b_*, *k_a_*, and *r_0_*, *φ_0_* are respectively bond and angle force constants and equilibrium values for bond length and angle.

The choice of parameters used in Eqs. 3–5 for the histone octamer, DNA, and histone-DNA bonds is described below.

### Histone Octamer

In the core part of the histone octamer each amino acid is represented as a single bead. The initial coordinates of the beads (of total number 710) were set to the centres of mass of the amino acids determined from the NCP high resolution crystal structure (entry code 1KX5.PDB, resolution 1.9 Å [2]). In order to keep the structure of the core region intact during simulations, while allowing for fluctuations, each bead of the core was connected to the three nearest beads by harmonic bonds, with the equilibrium distance equal to the distance between the amino acid centres of mass in the crystal structure and the force constant *k_b_* = 5·*kT/*Å^2^. This force constant results in bond fluctuations ±0.5 Å around the average positions, which corresponds to fluctuations of atomic groups in atomistic molecular dynamics simulations.

For each histone bead, a charge was assigned according to the amino acid type: lysine and arginine amino acids were given charge +1e, aspartate and glutamate charge was –1e, while the others were neutral. This resulted in 116 positive, 64 negative, and 530 neutral particles, with a total net charge +52e for the histone octamer (HO) core region.

The histone tails were modelled as 10 strings of linearly-connected beads of the length and amino acid sequence according to the PDB entry 1KX5.PDB [2]. The number of particles in each tail was 20, 35, 43, 24 and 13 for the H2A, H2B, H3 H4 and H2A-C (H2A-C is the short unstructured tail at the C-terminal of the H2A histone). The charges were also assigned according to the amino acid sequence, resulting in total charge +94e from 10 histone tails. The coordinates of the first (closest to the histone core) particle of each tail were chosen to match the coordinates of the tail in the crystal structure of the NCP. The beads of the tails were connected by a harmonic bond potential with a force constant *k_b_* = 5·*kT/*Å^2^ and an equilibrium bond length of 3.25 Å. This distance corresponds to the average contour length of a peptide chain per monomer as observed in all-atom MD simulations [79], [84] (and Korolev et al, unpublished data).

The hard core radii for all histone octamer beads was set to zero, except for the charged beads of the tails, for which it was set to 0.6 Å. Thus the effective radius of HO core beads and neutral beads of the tails was 2 Å while the effective radius of the positively charged beads of the tails was increased to 2.6 Å.

In assigning sizes and bonds for particles representing histones, we compared with results of our published [79], [81], [84] and unpublished results of all-atom MD simulations of interactions between fragments of the histone H4 tail and DNA oligomers of 22 bp length. These studies reported that tails were highly flexible and that they exhibit multiple direct contacts between DNA and peptide atoms, including penetration of the tails into the DNA grooves. Other all-atom MD simulations of the NCP (e.g. from Langowski laboratory [43], [85]) found that histone tails folded back to and interacted with the DNA and the histone core. Furthermore, in NCP crystals, partially resolved histone tails interact with DNA and with amino acids of the core histones in its own and with neighbouring NCPs. Based on this data we assigned rather small effective size for the charged and neutral particles to account for such direct contacts and to describe charge shielding and bridging functions of the tails.

### DNA

**Figure 3:**
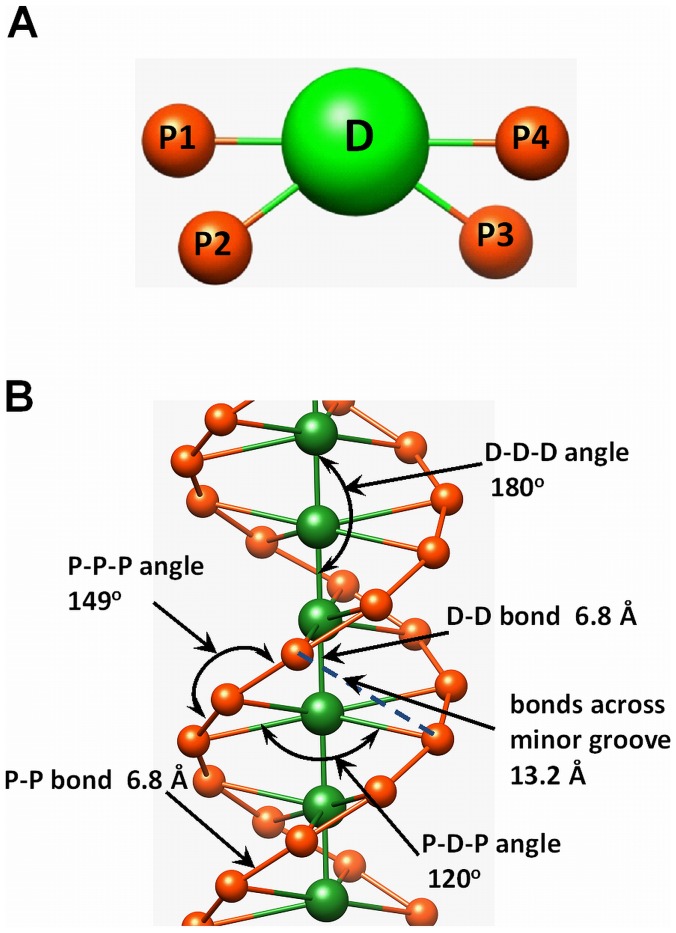
Coarse-grained model of DNA. (**A**) A 5-site two base pair elementary DNA unit where the central particle (green) approximates the pentose and base atoms of DNA and the four negatively charged particles (orange-red) are the phosphate groups at the positions of idealized B-form double helix. (**B**) Coarse-grained model of double helical DNA with bonds and angles set to maintain the B-form conformation (for details see the “DNA” section of Methods).

DNA was modelled as a sequence of 74 units each representing a two base pair fragment of DNA so that the total DNA length wrapping the histone octamer was 148 bp. Each unit consisted of five beads, four of which represent the phosphate groups (P) and one corresponding to the four bases in between (D) (Fig. 3A). The equilibrium geometry of the beads corresponds to the B-form of DNA (Fig. 3B). Each unit was connected to the previous one along the DNA axis with a distance between the central beads 6.8 Å and with a twist of the phosphate groups by 72° degrees, thus the whole structure forms a helical DNA with two clearly distinguishable strands of phosphate groups and minor and major grooves between them (Fig. 3B).

The integrity of the overall DNA structure was maintained by several harmonic bond and angle potentials described in Table 1. Each phosphate group was assigned a charge –1e. The hard core radii were set to 1 Å and 4 Å for phosphate groups and for DNA central beads respectively, resulting in effective radii 3 Å and 6 Å for phosphate and central beads respectively. The overall DNA structure thus resembles space-filling grooved DNA models used previously to model the ionic environment of DNA [74]–[77], [82].

**Table 1:** Parameters used in the coarse-grained model of double helical DNA.

Bonds/angle	Equilibrium distance/angle	Force constant	Comments
D-P*	9.62 Å	25* kT*/Å^2^	Phosphate- central bead bond
D-D	6.8 Å	25* kT*/Å^2^	Bond between neighbouring central beads
P1–P2; P3–P4; P2–P′1; P4-P′3	6.75 Å	25* kT*/Å^2^	Bonds connecting neighbouring phosphates along each strand
P1–P′3; P2– P′4	13.2 Å	5* kT*/Å^2^	Bonds across minor grove
D-D-D	180°	50* kT*/rad^2^	Keeps DNA backbone
P1-P2-P′1; P2-P′1-P′2; P3-P4-P′3; P4-P′3-P′4	149°	100* kT*/rad^2^	Angle along phosphate strands
P1-D-P3; P2-D-P4	140°	100* kT*/rad^2^	Keeps angle between the two phosphate strands

Particles are marked as in Fig. 3A: phosphates P1 and P2 belong to one DNA strand; P3 and P4 belong to the other strand; P′1–P′4 are phosphates from the next unit (see Fig. 3).

The described DNA model was linked to the histone octamer core by harmonic bonds connecting each DNA central bead D with the nearest amino acid bead of the histone core according to the crystal structure [2]. The force constant for these bonds was set to 5 kT/Å^2^. The histone tails protruded out from the core-DNA complex (Fig. 2). In total, the coarse-grained NCP model, consisting of the histone core, histone tails and DNA, includes 1350 particles with the net charge –150e.

We have tested the above coarse-grained DNA model in simulations of a 150 bp fragment in the presence of a monovalent electrolyte at 0.01 M concentration. As expected, through the simulations, DNA behaved like a wormlike chain with a persistence length of a few hundred Ångströms that maintained the correct double-helix structure. In the present work we did not pursue further optimization of DNA parameters since in the NCP simulation the DNA was all the time wrapped around the histone core. The approximation of the NCP as a rather rigid structure with limited fluctuation for DNA and the globular part of the HO is well-supported by experimental data. For example, in electron microscopy pictures of NCPs forming liquid crystals and aggregates reported by Livolant and co-workers [20], [22], [24], NCPs with deviating structures (e.g. with DNA ends detached from the particle) cannot be seen. Furthermore recent data on Förster resonance energy transfer spectroscopy reported that DNA in the NCP is typically fully wrapped around the HO and short fragments of about 10 bp experience short-lived detachment events (maximum 1–10% of the time; see e.g. work by Widom and co-workers [86]–[88] (and references cited therein). The model and parameter choice was validated by comparison with conformational properties obtained from small angle X-Ray Scattering (SAXS) data (see Results section below).

### Langevin Molecular Dynamics

The Langevin molecular dynamics method as implemented in the ESPResSo package [89] was used for all simulation of this work. This method, in addition to conservative forces defined by the force field expressions (1)-(5), includes a friction force -γ*ν_i_* (where γ is a thermostat friction parameter and *v_i_* is the particle velocity) and a random force, the intensity of which is determined by the fluctuation-dissipation theorem [90]. The electrostatic interactions were treated by the P3M method [91]. The friction and random forces mimic the effect of solvent, which makes the method very suitable to simulate continuum solvent models [64], [69]. Masses were given in reduced units and were set to 1 for all beads except the beads of DNA backbone, for which the masses were set to 4. Since we are interested only in equilibrium properties, we set the friction parameter to a low value, γ = 0.01, which results in faster sampling of the configuration space and a more efficient computation of the canonical averages.

The time step was set to 0.01 in the chosen (reduced) units, and we have made totally 10^8^ MD steps for a single-NCP simulations and 2.5^.^10^9^ MD steps for simulations with 10 NCPs, of which the initial 10^9^ MD steps were disregarded. Convergence of the simulations was checked by calculations of NCP-NCP centre of mass radial distribution functions (RDFs) in 2.5·10^8^ MD steps windows and ensuring that no trends in RDFs are observed.

### Spatial Distribution Function (SDF)

The results of the simulation with respect to the distribution of mobile ions and flexible histone tails as well as other NCPs around a given NCP in the simulation cell can be visualized using three-dimensional spatial distribution functions (SDF) applying an approach developed earlier for all-atom MD simulations of DNA [78], [92], [93]. The local coordinate frame for SDF calculations was determined from the positions of three selected sites in the distant parts of the globular part of histone octamer. Three-dimensional spatial distributions of the relevant particles around NCP were calculated in this frame which was also used for determination of the average NCP structure. SDFs were visualized as iso-surfaces drawn at the given level of intensity around average NCP structures determined in the same local frame.

Additionally, in order to provide a 3D impression of the spatial distribution of the mobile cations and flexible histone tails as well as to illustrate the average relative positioning of the NCPs in the NCP-NCP stacking interactions, visualization of the SDFs was used for creation a number of animations, which are available for downloading as Supporting Information.

## Results and Discussion

### Validation and Refinement of the NCP Model

We may note that the internal and histone DNA parameters were assigned based on choices to preserve a well-defined NCP structure that still allows for fluctuations around equilibrium positions and using our previous experience and information from literature (see above in the Materials and Methods section). Refinements and validation of the model by comparison with available experimental data was then performed as described below.

The choice of the bond force constants (*k_b_* = 5 kT/Å^2^ for the histone beads and for the DNA particles as listed in Table 1) was optimized by running several simulations with variation of the force constant and of the number of protein-protein (in the histone core) and core histone-DNA bonds. The root mean square deviation (RMSD) values between the HO structure and the initial HO model generated from the 1KX5 crystal structure have been monitored during the course of the simulations. It was found that after initial fast adjustment, the HO structure (excluding flexible histone tails) remains stable with standard deviation of RMSD being below 1 Å (see Figure S1). The initial instantaneous RMSD shift of the HO core was about 4–5 Å.

We also confirmed that the local structure of DNA remains preserved in comparison to the parameters that were set to mimic a canonical B form. For the system of a single NCP, we computed the distribution of distances for some pairs of DNA beads, with the following results (the values in parentheses refer to ideal B form): i) between opposite phosphates (same base pair): 18.0 (18.2) ±0.6 Å; ii) between neighbouring phosphates of the same strand: 6.6 (6.3) ±0.5 Å; iii) between DNA cores: 6.6 (6.8) ±0.5 Å. These distance distribution results are shown in Figure S2. These average distances show that the model of the DNA is robust, while still allowing for fluctuations. The distance between DNA cores is a bit smaller than “canonical” B form (3.4×2 = 6.8 Å) but due to DNA being wrapped around the histone core, some shortening of this distance can be expected.

A major aim of the present study is to develop a NCP model to be used in computer modelling with explicit mobile ions, and that can reproduce the self-association behaviour of NCP solutions in the presence of salt with varying counterion valence as demonstrated in experimental studies. Such investigations have demonstrated aggregation induced by different cations such as Mg^2+^ and CoHex^3+^ [28] and established the structural features in the formation of stacked NCPs in columnar phases using X-Ray diffraction and cryo-EM [19], [20], [22], [23], [94]. The results that are presented and discussed below gives very strong support for the present NCP model and its parameterisation and accounts exceptionally well for the structural details of stacked NCPs in the presence of multivalent cations.

In this section we discuss the analysis of the (monovalent) salt dependent structural features of individual NCPs in solution by comparison with available SAXS data, studying the effects of increase of salt on histone tail extension. Livolant and co-workers studied the effect of salt and role of histone tails on nucleosome conformation using solution SAXS [19], [25]. From the experimental scattering curves, the distance distribution, P(r) that represents all distances that can be found within the particle was calculated in the presence of varying NaCl salt. The data enabled the extraction of the maximal dimension of the particle, D_max_, as well as R_g_, the radius of gyration. D_max_ essentially measures the maximal separation between flexible tails and is expected to increase with salt as the tails extend and become more loosely associated with the core of its host particle. The most recent study on well-defined recombinant NCPs [19] demonstrated a modest increase in R_g_ from about 41.5 Å at low salt to 43 Å at high salt, while a corresponding increase in D_max_ from about 115 Å to 135 Å was observed.

We performed simulations calculating P(r) for a single NCP at different salt concentrations in the range 0–400 mM monovalent salt. The computational details are presented in the Supporting Information figure legend together with curves comparing P(r) for different concentrations of supporting salt in the solution (Figure S3). Table 2 shows the calculated values of these parameters and illustrates that the magnitude of D_max_ as well as R_g_ increase upon increase of salt concentration is in very good agreement with experimental observations (given the experimental uncertainty of about ±7%). It is reassuring that this particle conformational property agrees well with the experimental data as it reflects the tail extension and the flexibility of the tails; an important property for NCP-NCP interactions. The quantitative agreement is very reasonable given the experimental uncertainty in these measurements (about 5–10 Å variation). The simulated values (in particular R_g_) are somewhat smaller than the experimental ones obtained by the Livolant group. A quantitative variation of such a magnitude is not surprising due to three differences that are expected to shift R_g_ (and D_max_) to higher values compared to the simulations. Firstly, it has been shown that about 10 bp of DNA at the NCP entry/exit may dynamically detach from the NCP and these NCP “breathing” events become more frequent with increase of salt [86]–[88] (and references cited therein). This should shift the distributions towards larger distances compared to the simulations where these events do not occur. Secondly, in the cited experiments it has been shown that increase of the salt concentration leads to more frequent NCP-NCP interactions. These interactions allow additional tail extension as tails bridge neighbouring NCPs. This extension is expected to increase D_max_ and R_g_. In our system with one single NCP, tail bridging is not possible. Thirdly, even a small fraction of NCP aggregates (consisting of dynamic aggregates of two or more NCPs) in the experiments, would increase R_g_ and D_max_ while still being undetectable in the scattering profiles.

**Table 2:** Determination of R_g_ and D_max_ in the systems with single NCP and various concentration of added KCl salt.

Salt concentration, mM	R_g_, Å	D_max_, Å
Salt-free	39.1	115
50	39.2	116
100	39.3	118
400	39.8	128

**Figure 4:**
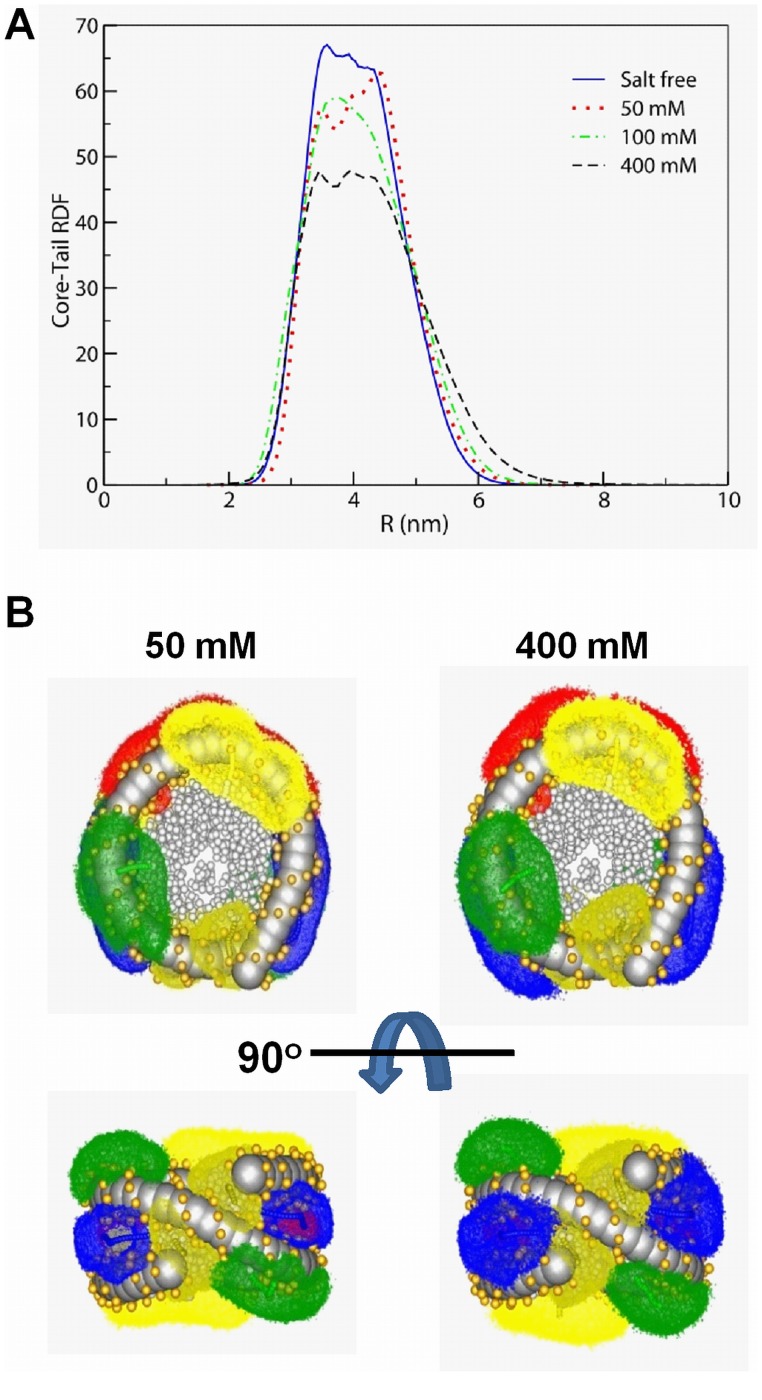
Salt dependence of the flexible histone tail attachment to the central fixed part of the NCP. (**A**) Tail-core RDFs calculated in systems with one NCP and different concentrations of KCl (indicated in the figure). (**B**) Comparison of the tail distribution visualized as SDFs for systems with one NCP and 50 mM (left) or 400 mM (right) of added KCl. Colour coding: H2A, N-termini, yellow; H2A, C-termini, dark yellow; H2B, red; H3, blue; H4, green; phosphate particles, gold.

The tail extension as a function of salt concentration that highlights the major effect of salt on the NCP conformation can be illustrated by tail-core RDFs (Fig. 4A) and SDFs (Fig. 4B). The increased intensity of the RDF for larger radial distances shows that tails extend when the salt concentration is increased (Fig. 4A). This behaviour is further demonstrated in Fig. 4B, which presents tail core SDFs, comparing the single NCP system at 50 and 400 mM of added salt. Tail extension observed with increase of salt is additionally illustrated in Figure S4.

## General Overview of Simulation Results

The present NCP model is a good representation of the detailed shape and electrostatic charge “mosaic” of the real NCP; including the specific structure of the electric field from the double helical B-form of DNA, the charge distribution in the histone tails and the existence of charged patches in the globular part of the histone octamer [1], [4]. The model also reflects the mobile and flexible nature of the unstructured histone tails, and it preserves the major advantage of our earlier NCP and nucleosome array models including the account of long-range electrostatics with explicit mobile ions.

We have carried out a series of Langevin molecular dynamics (MD) simulations of this NCP model in the presence of monovalent, divalent, and trivalent counterions representing K^+^/Na^+^, Mg^2+^ and cobalt(III)hexammine^3+^ (CoHex^3+^) cations. In order to limit the number of particles and to reduce computation time the systems contains no added salt, but only the neutralizing counterions. From the counterion concentration at the maximal distance from the centre of the NCP (where the electric field is approximately zero), it can be estimated that the present systems roughly corresponds to experimental situations of NCP solutions with K^+^, Mg^2+^ and CoHex^3+^ salts at “bulk” concentrations in the range 5–12 mM. This makes the systems comparable to experimentally studied NCP solutions precipitated by the presence of divalent and trivalent cations [20], [29]. Table 3 shows the number of particles in the simulated systems performed for these three types of counterions with one or ten NCP particles in a cubic simulation cell. The systems with ten NCPs correspond to dense solutions with NCP concentrations similar to that used in experimental X-ray diffraction and cryo-EM work by the Livolant group. Simulations of a single NCP have been performed in order to investigate properties of an “unperturbed” ionic atmosphere around an NCP not influenced by short-ranged NCP-NCP interactions.

**Table 3:** Number of particles used in the MD simulations.

System abbreviation	NCP	K^+^	Mg^2+^	CoHex^3+^	Cell size, nm
1 NCP/K^+^	1	150	0	0	40
1 NCP/Mg^2+^	1	0	75	50	40
1 NCP/Co^3+^	1	0	0	50	40
10NCP/K^+^	10	1500	0	0	40
10NCP/Mg^2+^	10	0	750	0	40
10NCP/Co^3+^	10	0	0	500	40

**Figure 5:**
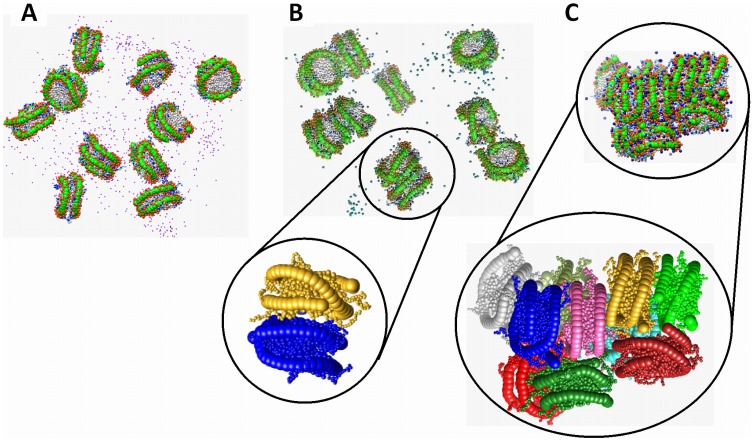
NCP-NCP interaction is defined by the valency of cation neutralizing NCP charge. Snapshots of the 10NCP/K^+^ (**A**), 10NCP/Mg^2+^ (**B**), and 10NCP/Co^3+^ (**C**) systems. Inserts below (**B**) and (**C**) illustrate close NCP-NCP interactions with the formation of stacked NCP pairs in the 10NCP/Mg^2+^ system and a dense NCP aggregate in the 10NCP/Co^3+^ system with combination of different types NCP-NCP contacts with stacking being dominant (note two NCP stacks, green-yellow and pink-blue forming a column). In the inserts, mobile cations and DNA phosphate groups are omitted for clarity.

Typical snapshots of the simulated systems with ten NCPs are shown in Fig. 5. It can be seen that in the presence of low concentration of monovalent K^+^ ions, the NCPs are well dissolved and behave as separate particles in solution. In the case of divalent Mg^2+^ counterions, the NCPs occasionally form clusters of 2–4 particles mostly contacting each other in a “stacking” fashion. For the case of three-valent spherical CoHex^3+^ cations, all NCPs assemble in a single compact cluster. The aggregation behaviour of NCPs is qualitatively similar to the experimentally observed properties of NCP solutions in the presence of low concentrations of Na^+^ (or K^+^), Mg^2+^ or CoHex^3+^ salts [17], [20], [28], [95]. Below we analyse and discuss data describing NCP-NCP interaction observed in the simulations. Furthermore, other results of the simulations will be presented and analysed.

### NCP - NCP Interactions

Characterization of both intra- and intermolecular NCP interactions between different components of the NCP can be made using various radial distribution functions.

**Figure 6:**
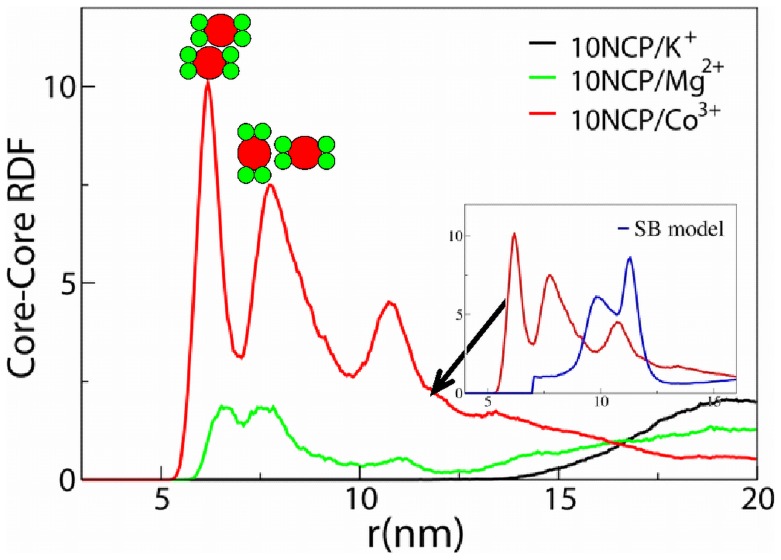
Radial distribution functions calculated for the centres of the NCP histone core. Schematic representations of the NCP-NCP contacts are shown above the corresponding RDF peaks. In the insert, core-core RDF determined for the new coarse-grained NCP model in the 10NCP/Co^3+^ setup is compared with the RDF in the similar 10NCP/Co^3+^ system from our earlier work [28] where the histone core was modelled as single spherical particle (blue curve).

The core-core RDFs are displayed in Fig. 6. The NCP centre in the core-core RDF calculation is defined as the centre of mass of the globular part of the histone octamer comprising 710 particles in the current NCP model; the particles from the histone tails and DNA are discarded. For the 10NCP/K^+^ system, the core-core RDF is equal to zero at distances less than 12 nm and does not show any maxima, indicating that in solution with monovalent counterions NCPs repel each other and do not form close contact. This result is in agreement with experimental observations obtained for an NCP solution at low concentration of monovalent salt [18], [29] and with our earlier simulation work where a simpler NCP coarse-graining was used [28], [53], [64]. It would also be of interestto study if the new NCP model is able to capture the transition from repulsive to attractive NCP-NCP interaction with an increase of monovalent salt concentration observed in experiments [18], [29] and in the simulations with simpler NCP models [28], [53], [64].

In the presence of Mg^2+^ (10NCP/Mg^2+^ system) the formation of NCP clusters is reflected in the RDF by the appearance of three core-core correlation peaks which correspond to different relative positions of contacting NCPs shown as sketches in Fig. 6 (see below). This result agrees qualitatively with the experimental data [20], [28], [95] and with the earlier simulations [28], [53], [64].

For the three-valent CoHex^3+^, core-core RDF shows maxima similar to the 10NCP/Mg^2+^ system but of larger intensity. The first maximum at about 6 nm corresponds to a stacking coordination of NCPs when the flat surfaces of the histone cores are in close contact. These NCP stacks can be seen in snapshots in Fig. 5. The second maximum at about 8 nm represents NCP coordination where two flat NCP cylinders are approximately perpendicular to each other. The third maximum at 11 nm corresponds to the NCPs in the second coordination shell around close stacked contacts registered by the first RDF maximum. During the simulations, NCPs repeatedly changed their coordination and contacts, and the RDFs shown in Fig. 6 reflect averaged population of the corresponding core-to-core distances. Since convergence in the 10NCP/Co^3+^ system forming a single aggregate of 10 NCPs is slower than in the 10NCP/Mg^2+^ and 10NCP/K^+^ systems, the simulations for the former system were longer compared to the other two systems with 10 NCPs. Comparison of the core-core RDFs of the CoHex^3+^system calculated for different parts of the MD trajectory has shown similar profiles with some variation of relative intensity between three maxima that can be expected since dynamics of the NCPs inside the cluster is slow and the small number of NCPs limits sampling of all possible structures. It has to be also noted here that small number of NCPs in the simulation cell precludes formation of extended columnar structures observed in experiment [22], [24]. Convergence of the systems was checked by monitoring the core-core and other RDFs calculated for different time frames of the trajectories and showed no systematic changes in these average quantities reflecting the equilibrium properties of the system (data not shown).

The present model eliminates a drawback of our earlier more approximate modelling of the histone core as a spherical particle. Such an approximation of the histone core limits the formation NCP-NCP stacking contacts as illustrated in the insert of Fig. 6 where core-core RDFs of the two NCP coarse-grained models calculated for the 10NCP/Co^3+^ systems are compared. However, that more approximate model of the NCP highlighted the primary importance of the long-range electrostatic forces since it managed to capture the salt-dependent NCP-NCP aggregation and qualitatively reproduced experimental behaviour of NCP solutions and nucleosome arrays [53], [64], [69], [70]. The higher resolution of the present coarse-grained model allows us to investigate the geometry and the interactions responsible for the formation of the NCP-NCP stacking, which is discussed below.

### NCP Interaction with Mobile Ions

**Figure 7:**
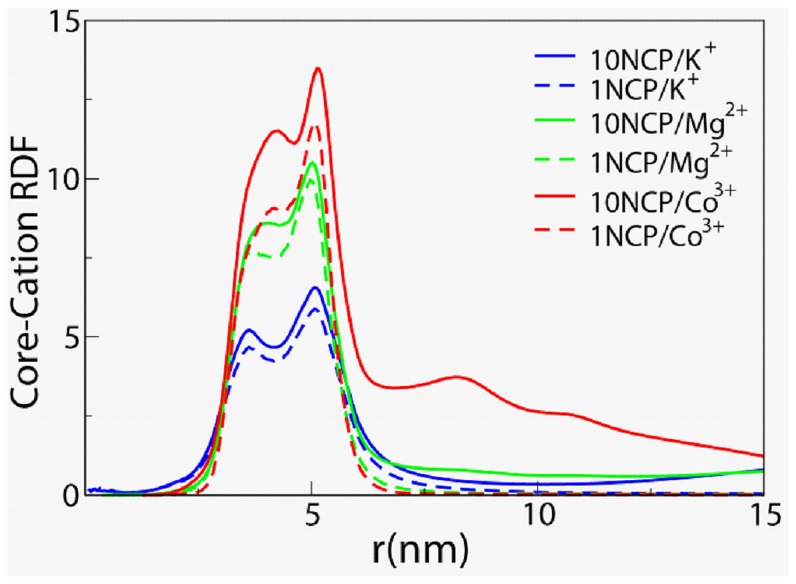
Radial distribution functions between NCP core and K^+^ (blue), Mg^2+^ (green) and CoHex^3+^ (red) cations in the systems with ten (solid curves) and single (dashed lines) NCP.

RDFs between NCP core and counterions are shown in Fig. 7. Convergence of the cation-NCP interaction is fast in all simulated systems. All core-ion RDFs have a similar shape with two maxima at about 3.6 and 5.2 nm. The first maximum is formed by the counterions interacting with DNA phosphates situated closer to the histone core whereas the second maximum is due to cation attraction with the DNA phosphates located on the outside surface of the NCP wedge-shaped cylinder. RDFs of the cations relative to the DNA phosphates are displayed in the Figure S5A. The cation concentration close to the NCP (the first and second maxima) increases with increase in the valence of the counterions. For the systems with a single NCP, the cation density is significantly diminished at large distances from the NCP which indicate that most counterions are concentrated near the NCP and that their concentration in the “bulk” (even for the monovalent ions) is very small, an expected polyelectrolyte behaviour. In the simulations with ten NCPs where we have the same box size, the NCP concentration is considerably higher, a similar steep decay in the observed RDFs is not be expected and indeed not observed due to the high counterion concentration and relatively small box size for these systems (compared to a single NCP). This relatively small box size is a natural choice as it is similar to experimental studies of dense NCP solutions and is also computationally efficient. In the 10NCP/K^+^ system, the average separation between NCPs is around 20 nm and a minimum of the negative electric field occurs at around 10 nm from the centre of mass of each NCP; at larger distances the increase of cation concentration indicates the presence of other NCPs in the cell. For the case of monovalent salt the effective interaction between NCPs is repulsive and NCPs do not come close to each other (see also the core-core RDF in Fig. 6). For the CoHex^3+^ system there is a significantly less steep decay of the intensity of the RDFs at large distances and this is a result of the formation of one large cluster of NCPs and reflects the short average separation between such clusters in neighbouring cells (from the periodic images in the simulation).

In the 10NCP/Co^3+^ system, the core-CoHex^3+^ RDF shows a high amplitude within a 6–12 nm range caused by counterions associated with other NCPs inside the single cluster formed by all NCPs. Clustering of the NCPs in aggregates of 2–4 NCPs observed in the 10NCP/Mg^2+^ system is reflected only by a small “bump” of the Mg^2+^ density at 8–9 nm from the NCP core. Comparing RDFs in the systems with a single NCP and with ten NCPs, one can note that counterions from neighbouring NCPs contribute more to the first than to the second RDF maximum, which is most noticeably pronounced for the case of CoHex^3+^. When a “stacking” conformation is formed, counterions which are present in the contact region with histone core become shared between two NCPs and thus contribute to the first RDF maximum.

### NCP Interaction with Internal and External Histone Tails

**Figure 8:**
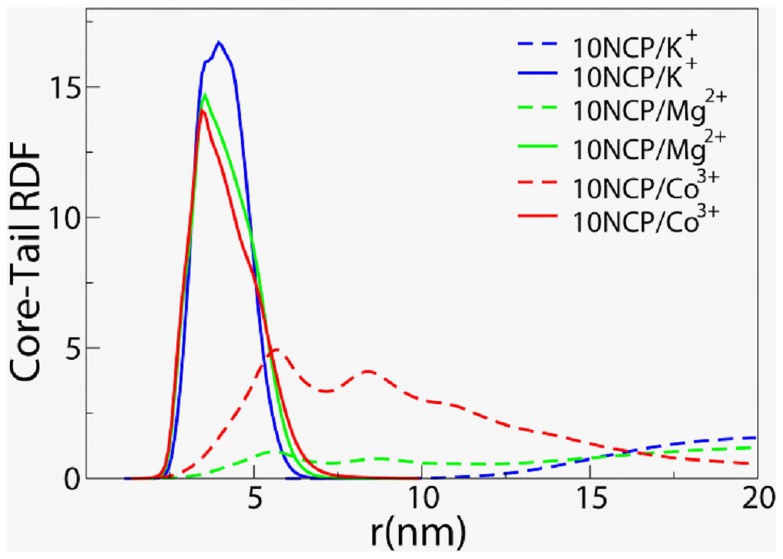
Radial distribution functions between the NCP core and internal (solid curves) or external (dashed lines) histone tails in the systems of ten NCPs with K^+^ (blue), Mg^2+^ (green) and CoHex^3+^ (red) cations.

RDFs between the NCP core and the histone tails belonging to the “host” NCP (shown as solid lines) and the external tails (dashed curves) in the systems with ten NCPs are shown in Fig. 8. In all systems (both with one and with ten NCPs) the internal tails are confined in close vicinity of the histone core and interact with the DNA phosphates (see Figure S5B of the Supporting Information). The tails with exit points located between the DNA gyres (H2A/C, H2B and H3), remain locked into the narrow space between the two DNA helices on the lateral surface of the NCP (see also next section describing spatial distribution functions and for 3D impression see animation Movie S1). The tails stemming from the top and bottom of the NCP wedge-shaped cylinder (H2A and H4) interact with the DNA phosphates they can reach. The higher the valency of the mobile cation, the more potent is the cation to compete with the tails for binding to the DNA and that is reflected by a decrease in the maximum of the core-tail RDF following the order 10NCP/K^+^ <10NCP/Mg^2+^<10NCP/Co^3+^ (Fig. 8). Since the most frequent binding sites for the mobile cations and the positive beads in the histone tails are the DNA phosphates, the histone-core and the histone-phosphate RDFs show a similar trend (Figure S5B and Figure S6).

Non-zero values of the external tail-core RDFs observed at 3–8 nm distance in the 10NCP/Mg^2+^ and 10NCP/Co^3+^ systems indicate participation of the tails in the NCP-NCP interaction (Fig. 8, dashed lines). Generally, the external or ‘foreign’ tails are not capable of penetrating and substituting the NCP tails at the positions with the highest negative electric field (between the DNA gyres on the NCP lateral surface or in the pockets between the histone core and DNA). The external tails interact mostly with the DNA phosphates distant from the histone core. A relatively small fraction of the tails makes bridges with neighbouring NCP in the 10NCP/Mg^2+^ and 10NCP/Co^3+^ systems, which can be seen by comparing intensities of the external and internal tail-core RDFs in Fig. 8. Consequently, it can be concluded that a major influence of the positively charged tails to the NCP-NCP attractions comes from their screening of the DNA negative charge.

In the condensed 10NCP/Co^3+^ system, the tails of the H4, H2A and H3 histones can make bridges to the neighbouring nucleosomes. (See Figure S7 displaying external tail-core RDFs calculated for each type of tail in the 10NCP/Co^3+^ and 10NCP/Mg^2+^ systems). Analysis of spatial distribution functions, SDFs, shows that the H4 and H2A tails mediate NCP-NCP stacking by forming an organized network of histone bridges between adjacent NCP surfaces at the top and the bottom of the NCP cylinders (see also next section and animation Movie S2 (10NCP/Mg^2+^ system) and Movie S3 (10NCP/Co^3+^ system)). The H3 tails makes most of the contacts with the DNA on the lateral surfaces and these contacts are more diffuse so they don’t show areas of high density in the SDFs. Lesser involvement of the H3 tail (compared to the H4 and H2A tails) to NCP-NCP bridging is observed in the 10NCP/Mg^2+^ system.

**Figure 10:**
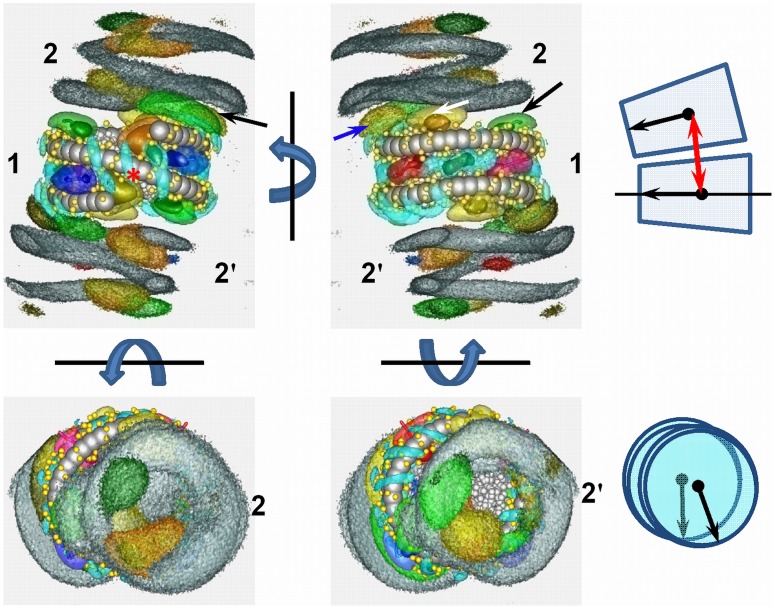
NCP-NCP stacking in the 10NCP/Co^3+^ system. Formation of ordered NCP structures is visualized by displaying SDFs of *external* DNA and histone tail calculated relative to the central nucleosome (NCP-1). SDFs of CoHex^3+^ (cyan) and *internal* histone tails are also shown. The two top panels present views facing the NCP dyad axis (left, position of dyad axis is indicated by red star) and opposite view (right panel, 180° rotation along vertical axis). The two pictures at the bottom show SDFs seen from the top (left) and bottom (right) relative to the orientations shown at the top. Images of the SDFs calculated for external DNA highlight stable positioning of the two NCPs (numbered 2 and 2′) relative to the central NCP. The orientation of the NCPs is illustrated by 2 sketches to the right clarifying the nucleosome orientation in the stack: slightly shifted from a position of complete alignment and oriented close to perfect in a “head-to-head” orientation (as shown in the bottom-right sketch). The NCP-NCP interaction is stabilized by tail-tail correlations and tail bridging with participation of the H2A (yellow) and H4 (green) tails on each side of the NCP-1: Each tail forms a “shield” between DNA/histone core surfaces of interacting NCPs which does not overlap with its neighbour. For example, repulsion between NCP-1 and NCP-2 is screened by NCP-2 H4 tail bridging to DNA of the NCP-1 (red arrow in the top-left panel) followed by bridges: NCP-2 H2A tail – DNA NCP-1 (blue arrow, top-right panel), NCP-1 H2A tail – DNA NCP-2 (white arrow, top-right) and NCP-2 H4 tail – DNA NCP-2 (black arrow, top-right). The tails H2B (pink), H3 (blue) and H2A-C (yellow/brown) do not take part NCP interaction and their SDFs are confined close to their host NCPs. See also the animation Movie S3 (Supporting Information).

Experimental data obtained for some NCP crystals [1], [2], [4] and nucleosome arrays [34], [96] indicate that NCP-NCP interaction includes formation of contacts between histone H4 residues 16–23 and the negatively charged domain on the surface of the histone octamer formed by six acidic amino acids of the H2A histone. The negative charge density of this H2A acidic patch might form a binding site for the positive amino acids of the internal histone tails. We checked the presence of such histone tail – histone core interactions by calculating RDFs between internal or external histone tails with the respective amino acids of the H2A acidic patch (Figure S8 of Supporting Information). It was found that, in contrast to the tail-DNA phosphate interactions, direct contacts of both the internal and external tails with the acidic patch are practically absent, since no RDF maxima were observed below 2 nm. This difference between modelling and experimental data might be due to the imperfection of the present NCP model, which does not include attractive van der Waals and hydrogen bonding interactions. Another possibility is that the “head-to-head” orientation of the NCPs in the stacks (the “head” of the NCP is defined as position of the dyad axis; see Fig. 10 below and next section) observed in the present simulations and experimentally in liquid crystalline NCP phases [20], [23], [24] is different from the “head-to-tail” NCP-NCP orientation seen in most of the deposited crystal structures of the NCP (e.g. [1], [2]). Furthermore, the structure of the tetranucleosome showed a “head-to-head” NCP stacking and orientation of the neighbouring nucleosomes that precluded interaction between the H4 tail and the acidic patch [33].

### Spatial Distribution Functions (SDFs)

**Figure 9:**
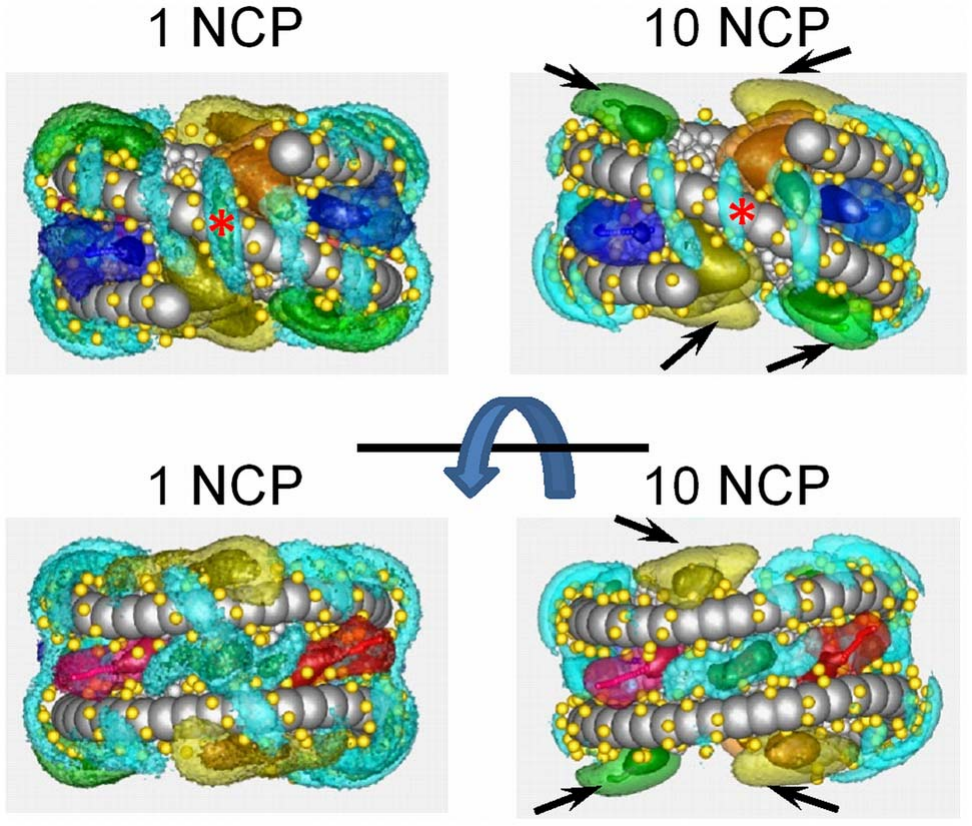
Comparison of SDFs calculated for the CoHex^3+^ (cyan) and *internal* histone tails in systems with one (two panels to the left) and ten NCPs (two right-hand side panels). Arrows in the right panels indicate extension of the H2A (yellow) and H4 (green) tails outside the histone core and DNA due to NCP-NCP interaction in the 10NCP/Co^3+^ system. SDFs of the H2B and H3 tails are shown respectively in pink and blue colours; particles representing DNA and histone core are in light grey; phosphates are yellow. Red stars show position of the dyad axis.

More insight into organization of the counterions and tails around NCP and about the structure of the NCP-NCP contacts can be gained from observations of the spatial distribution functions (SDFs). Figure 9 compares SDFs calculated for systems of one and ten NCPs with K^+^ and CoHex^3+^ (more data is shown in Figure S9). Additionally, to provide three-dimensional information about the distribution of ions, tails and NCPs, we provide movies available as Supporting Information: Movie S1 (10NCP/K^+^ system, internal SDFs), Movie S2 (10NCP/Mg^2+^ system, external SDFs), and Movie S3 (10NCP/Co^3+^ system, external SDFs).

The counterions K^+^ and CoHex^3+^ (shown in cyan) are concentrated in the area of a high negative electric field of the DNA around the minor groove. All tails are mobile and average coordinates of the tail beads do not give a good estimation for the typical tail locations. Therefore we computed SDFs also for tails in order to gain a better understanding of the tail spatial distribution. In agreement with the corresponding RDFs, all tails remain confined in the vicinity of their “host” NCP both in the systems with one and with ten core particles. Only for the 10NCP/Co^3+^ system, did the SDF visualization show some H4 and H2A tail extension outside their host NCP (tail extension is indicated by the arrows in Fig. 9). It is also interesting to note that SDFs of the tails and mobile cations do not overlap.

To further describe the NCP-NCP interaction in the system with ten NCPs, we calculated SDFs of the *external* tails and DNA (phosphate and base pair particles) relative to a central NCP. Data for the 10NCP/Co^3+^ system is shown in Fig. 10; similar projections for the 10NCP/Mg^2+^ system are given in Figure S10. As this analysis gives information on the average relative geometry of the nucleosome-nucleosome contacts, it can be used to investigate the nature of the stacking between nucleosome pairs. Remarkably, the strongly aggregated 10NCP/Co^3+^ system produced a well-defined average geometry for the NCP-NCP stacking “column”. The stacked NCPs are oriented “head-to-head” or “front-to-front” relative to the dyad axes and slightly shifted from the position of perfect overlap (see the explanatory information in Fig. 10; see also animation Movie S2 (10NCP/Mg^2+^ system) and Movie S3 (10NCP/Co^3+^ system). This results is in excellent agreement with electron microscopy and X-ray diffraction data obtained by Livolant group [20], [23], [24]. The cited papers reported “head-to-head” NCP stacking with an angle of 14° due to a shift between neighbouring NCPs from a position of perfect alignment. The NCP-NCP stacking distance estimated from the X-ray diffraction data is in a good agreement with the position of the first maximum in the core-core RDFs for 10NCP/Mg^2+^ and 10NCP/Co^3+^ systems (Fig. 6): about 6–7 nm in Fig. 6 compared to experimental values of 5.9–6.2 nm or 5.5 nm for NCPs condensed respectively by osmotic stress [23] or by Mg^2+^, spermidine^3+^ [20] or CoHex^3+^ (Y. Liu, N. Korolev, L. Nordenskiöld et al, unpublished data). Furthermore, the separation between columns of stacked NCPs (10.7–11.2 nm [23] or 11.3–12.0 nm [20]) agrees with the position of the third maximum at about 10.5–11.7 nm in the core-core RDF of the 10NCP/Co^3+^ system (Fig. 6). Interestingly, the X-ray diffraction data could not distinguish between the vertical, fully overlapped and “skewed” NCP stacking [23]. Only careful examination of electron microscopy images resulted in the conclusion that the NCPs were shifted relative to each other (with an angle 14°) and that the NCPs were in the “head-to-head” orientation. Precisely that sort of NCP-NCP stacking structure is most frequently observed in the 10NCP/Co^3+^ and 10NCP/Mg^2+^ simulations (Fig. 10 and Figure S10). Detailed analysis of the mutual orientation of the stacked NCPs in the 10NCP/Co^3+^ system showed that the distribution of angles between the dyad axes of the stacked NCPs has a broad maximum in the range 20–60° (data not shown) in agreement with the value of this angle (34°±4°) reported in a recent study [95] and about 26° observed in the crystal of the tetranucleosome [33]. This agreement between modelling data and the actual experimental observations provides very convincing validation of our NCP model.

Visualization of the NCP stacking showed that only the H4 and H2A tails take part in this inter-nucleosome interaction forming “a circular chain” of crosslinks inside the NCP-NCP overlapping area. While participation of the H4 tail in the NCP-NCP interaction is well documented and only this tail (one of the two) was detected between stacked NCPs in the crystal structures, a similar contribution from the H2A tail has not been reported. Additional explanations of the features illustrated by the SDFs are given in the figure legends.

### Conclusions and Perspectives

The present work introduces a new coarse-grained model of the nucleosome core particle which provides a detailed description of the nucleosome shape and the distribution of charged amino acids of the protein and phosphate groups of DNA. The simulations with counterions of different charge mimicking NCP solutions in the presence of explicit K^+^, Mg^2+^ and CoHex^3+^ ions produced a remarkably realistic picture of the NCP-NCP interaction. Not only does NCP-NCP attraction appear after substitution of K^+^ by Mg^2+^, and full condensation of the NCPs is observed in the presence of CoHex^3+^, but the geometry of the structure of the NCP-NCP stacking contacts closely matches the X-ray diffraction and electron microscopy observations [23], [24]. Furthermore, the present simulation setup maintains the major advantage of our earlier, coarser description of the NCP and nucleosome arrays, namely explicit presence of the mobile ions. Our previous simulations [28], [53], [64], [69], [70], [81] showed that inclusion of explicit ions into the model is critically important for physically sound description of the effect of multivalent ions on the aggregation behaviour of NCPs and NCPs array.

The present model maintains the integrity of the NCP by a number of harmonic bonds between the DNA and the histone core components whereas in the real NCP no such bonds exist between these components and DNA is held the core by electrostatic forces and by the formation of a large number of ionic and hydrogen bonds [1], [2]. In the future, development of this model can be obtained by employing more realistic effective potentials derived within the concept of multi-scale modelling, on the basis of atomistic simulations of the NCP components-DNA, histone amino acids. The process of such parameterisation may be long but would enable the inclusion of various effects of the molecular nature of the solvent such as hydration, hydrogen bonds, and other specific interactions not present in the current model. The description of solvent mediated electrostatic interactions (i.e. going beyond the continuum approximation) will improve the modelling of the electrostatic interactions and is desirable for a description of salt dependence of NCP-NCP interactions. Introduction of more realistic bonding and non-bonding potentials will allow modelling of dynamical properties of the nucleosome, that can enable description of the dynamics of DNA detachment from the histone core (“breathing” of nucleosomal DNA [85]–[88]) and mechanical unpeeling of DNA from the histone core as investigated in single molecule experiments [97], [98] (reviewed in [99]). It should also give even better agreement between the experimental (SAXS) and simulated salt-dependent NCP conformational properties.

However, even at the present stage, the new NCP model provides a realistic description and is consistent with experiment of NCP interactions at varying ionic conditions. In the future the present model can be used for studies of chromatin folding and its structural properties by introducing linker DNA, connecting separate NCPs into a nucleosome array.

## Supporting Information

Figure S1 **RMSD of the histone octamer compared to its starting configuration generated from the 1KX5 crystal structure, observed during the course of the simulation for a system with a single NCP and K^+^ counterions (1NCP/K^+^).**
(TIF)

Figure S2 **Distribution of distances between selected beads of DNA observed during the course of the simulation in the system of 1NCP/Co^3+^.**
(TIF)

Figure S3 **Comparison of the NCP particle distance distribution functions, P(r), for systems with a single NCP and with different salt concentrations: salt free (only K^+^ counterions), 50, 100 and 400 mM KCl.**
(TIF)

Figure S4 **Comparison of the tail spatial distribution functions (SDF) calculated for systems with 50 and 400 mM KCl concentrations and with one NCP in the 30-nm simulation box.**
(TIF)

Figure S5 **Comparison of mobile counterion distributions around DNA phosphate groups (A) and internal histone tail particles (B) in systems with one and ten NCPs.**
(TIF)

Figure S6 **External histone tail–phosphate RDFs calculated in simulations with ten NCPs and different counterions.**
(TIF)

Figure S7 **Radial distribution functions between the NCP core and external histone tails calculated separately for each tail in the 10NCP/Co^3+^ (A) and 10NCP/Mg^2+^ (B) systems.**
(TIF)

Figure S8 **Comparison of distribution of internal and external histone tail particles around the acidic patch of the histone core.**
(TIF)

Figure S9 **Comparison of the** ***internal*** **SFDs of the histone tails and the cations K^+^, Mg^2+^ and CoHex^3+^ calculated for systems with single and 10 NCPs.**
(TIF)

Figure S10 **NCP-NCP stacking in the 10NCP/Mg^2+^ system.** Formation of ordered NCP structures is visualized by displaying SDFs of *external* DNA and histone tails calculated relative to the central nucleosome.
(TIF)

Movie S1 **SDFs of counterions and** ***internal*** **histone tails in the 10NCP/K^+^ system.**
(MP4)

Movie S2 **SDFs of the counterions,** ***external*** **histone tails and SDF of external DNA in the 10NCP/Mg^2+^ system (averaged position of the** ***internal*** **tails is shown as small beads).**
(MP4)

Movie S3 **SDFs of counterions,** ***external*** **histone tails and SDF of external DNA in the 10NCP/Co^3+^ system (averaged position of the** ***internal*** **tails is shown as small beads).**
(MP4)
