# Supplementary figures and images for: An Advanced Coarse-Grained Nucleosome Core Particle Model for Computer Simulations of Nucleosome-Nucleosome Interactions under Varying Ionic Conditions
Source: PLoS One. 2013 Feb 13;8(2):e54228. doi: 10.1371/journal.pone.0054228 (PMC3572162; doi:10.1371/journal.pone.0054228)

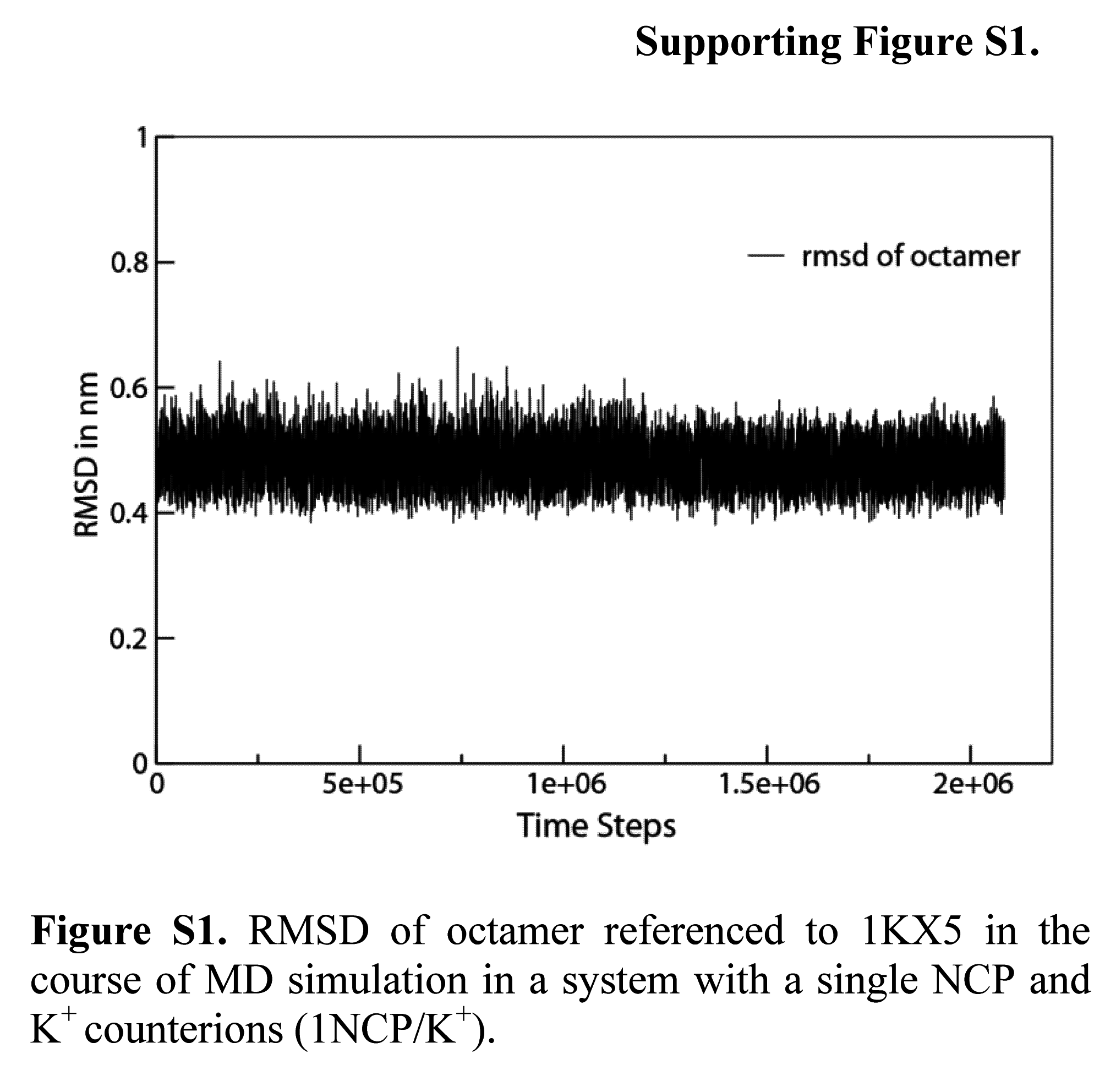

Supplement: Figure S1 — RMSD of the histone octamer compared to its starting configuration generated from the 1KX5 crystal structure, observed during the course of the simulation for a system with a single NCP and K+ counterions (1NCP/K+). (TIF) [file pone.0054228.s001.tif]

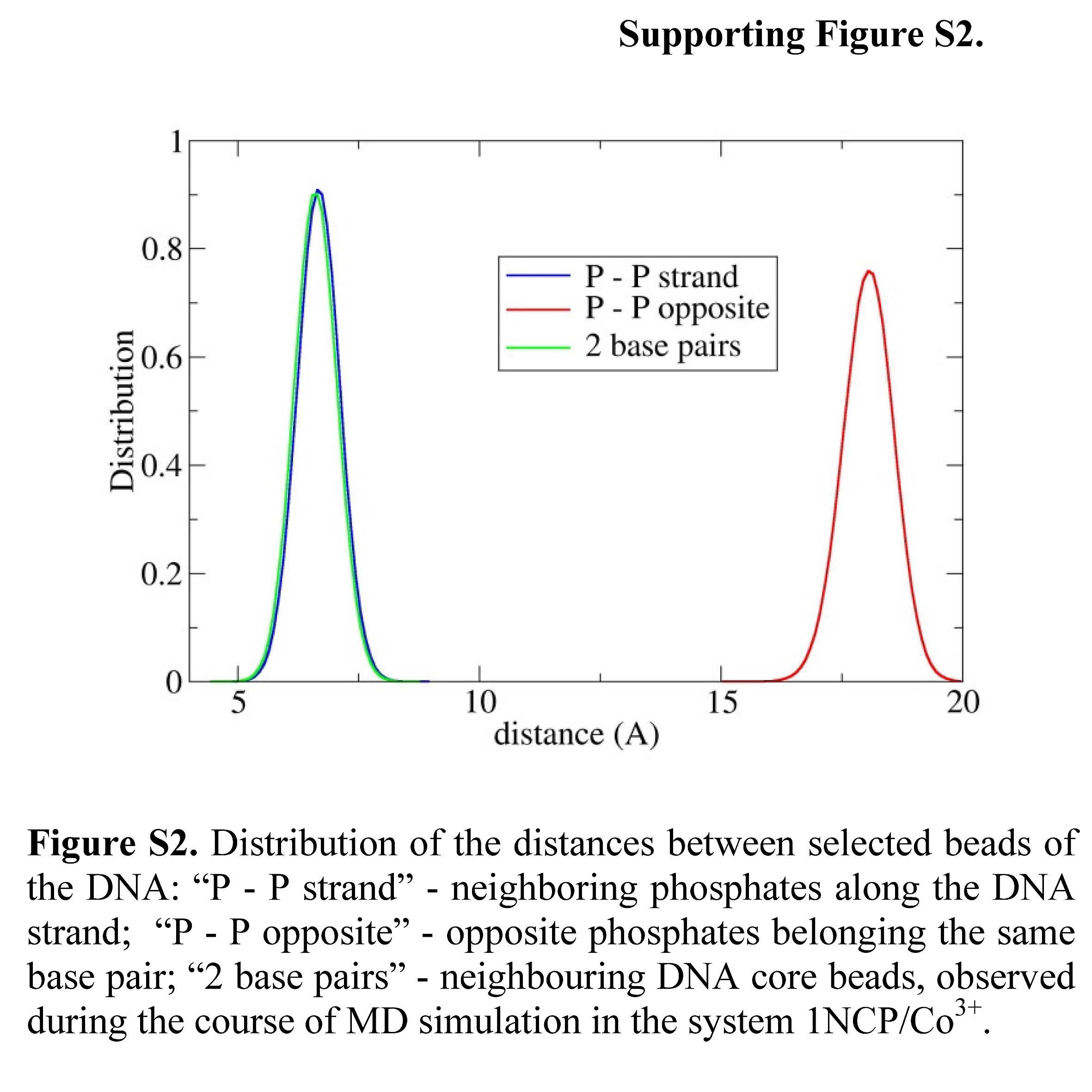

Supplement: Figure S2 — Distribution of distances between selected beads of DNA observed during the course of the simulation in the system of 1NCP/Co3+. (TIF) [file pone.0054228.s002.tif]

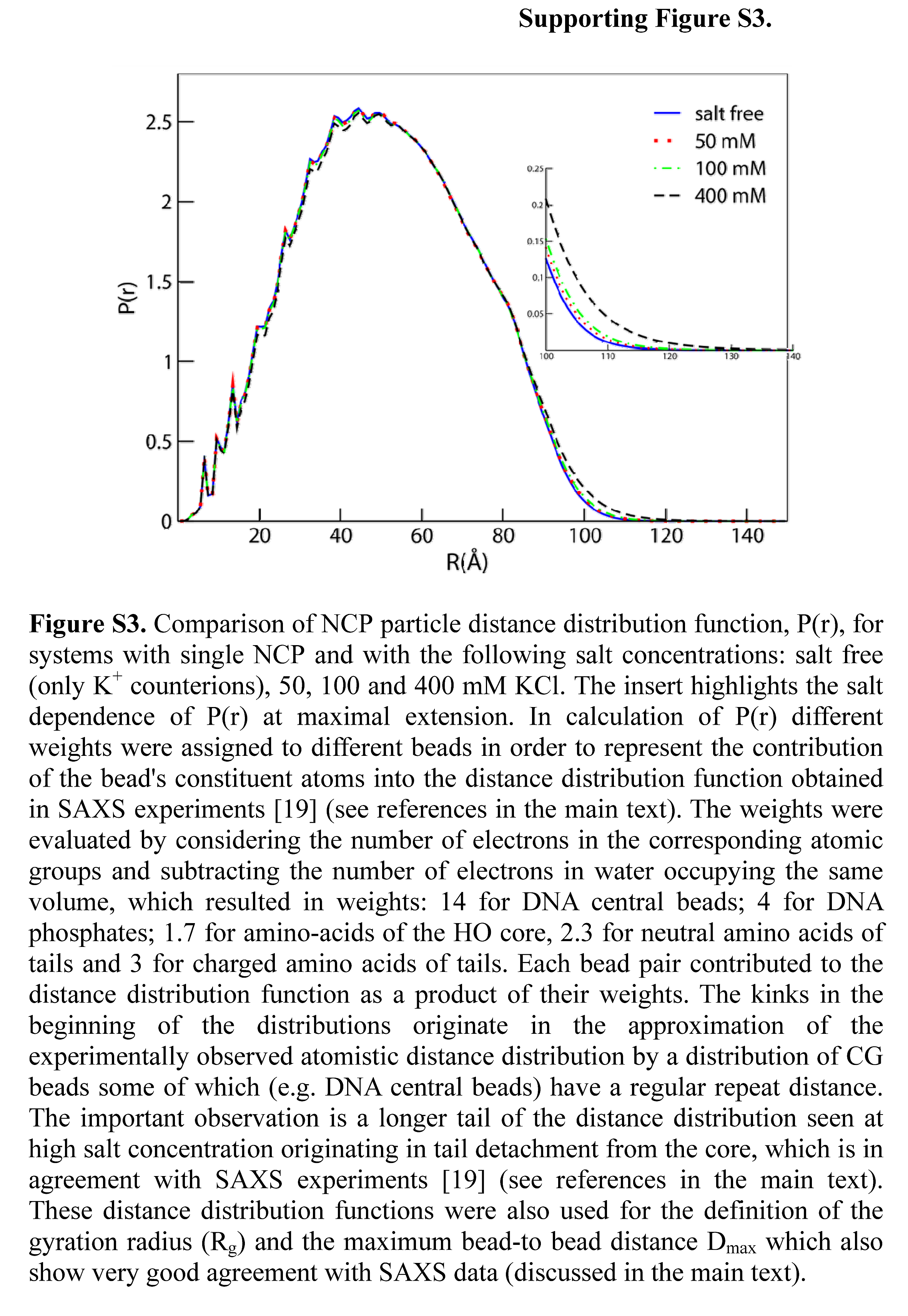

Supplement: Figure S3 — Comparison of the NCP particle distance distribution functions, P(r), for systems with a single NCP and with different salt concentrations: salt free (only K+ counterions), 50, 100 and 400 mM KCl. (TIF) [file pone.0054228.s003.tif]

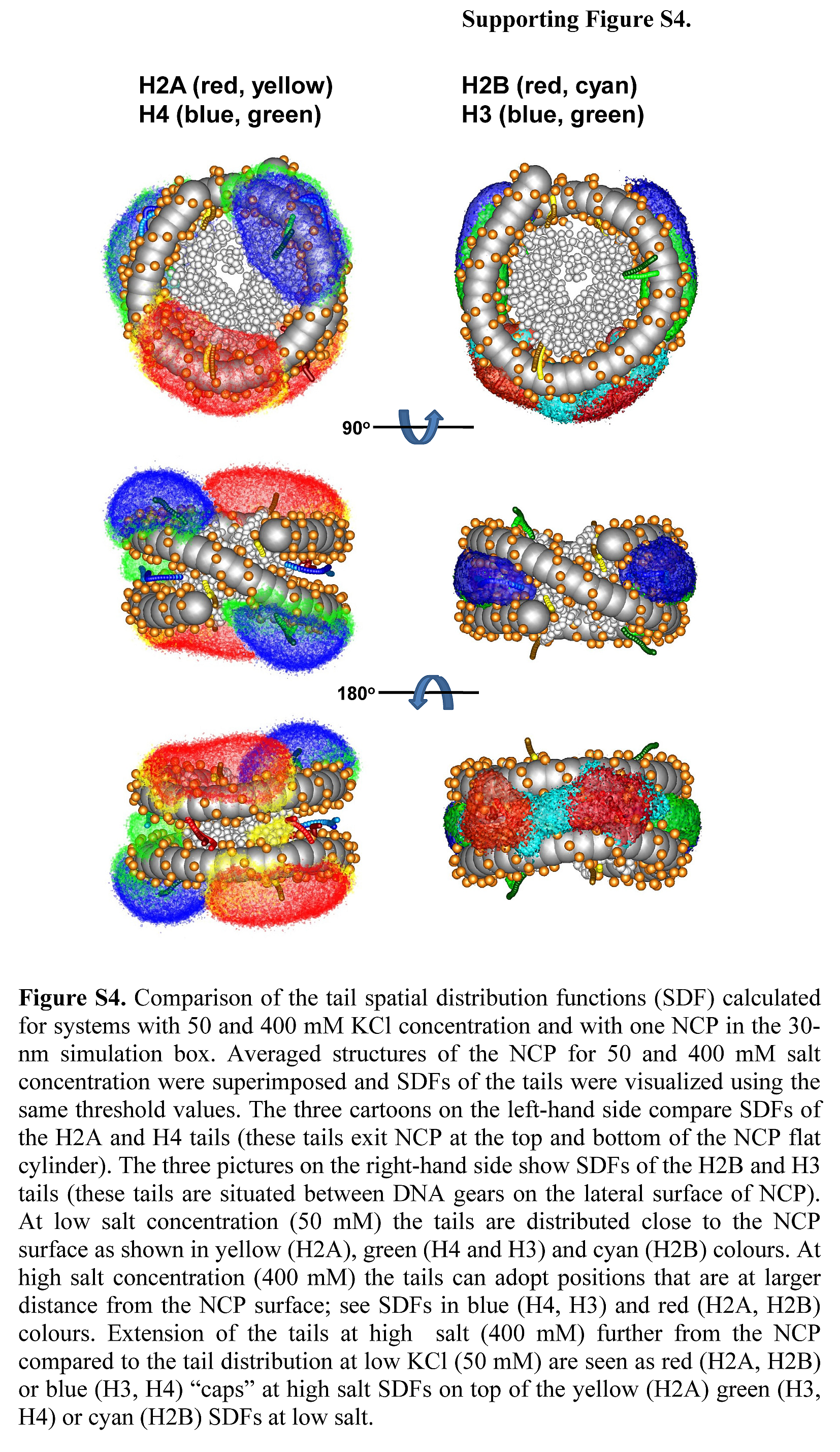

Supplement: Figure S4 — Comparison of the tail spatial distribution functions (SDF) calculated for systems with 50 and 400 mM KCl concentrations and with one NCP in the 30-nm simulation box. (TIF) [file pone.0054228.s004.tif]

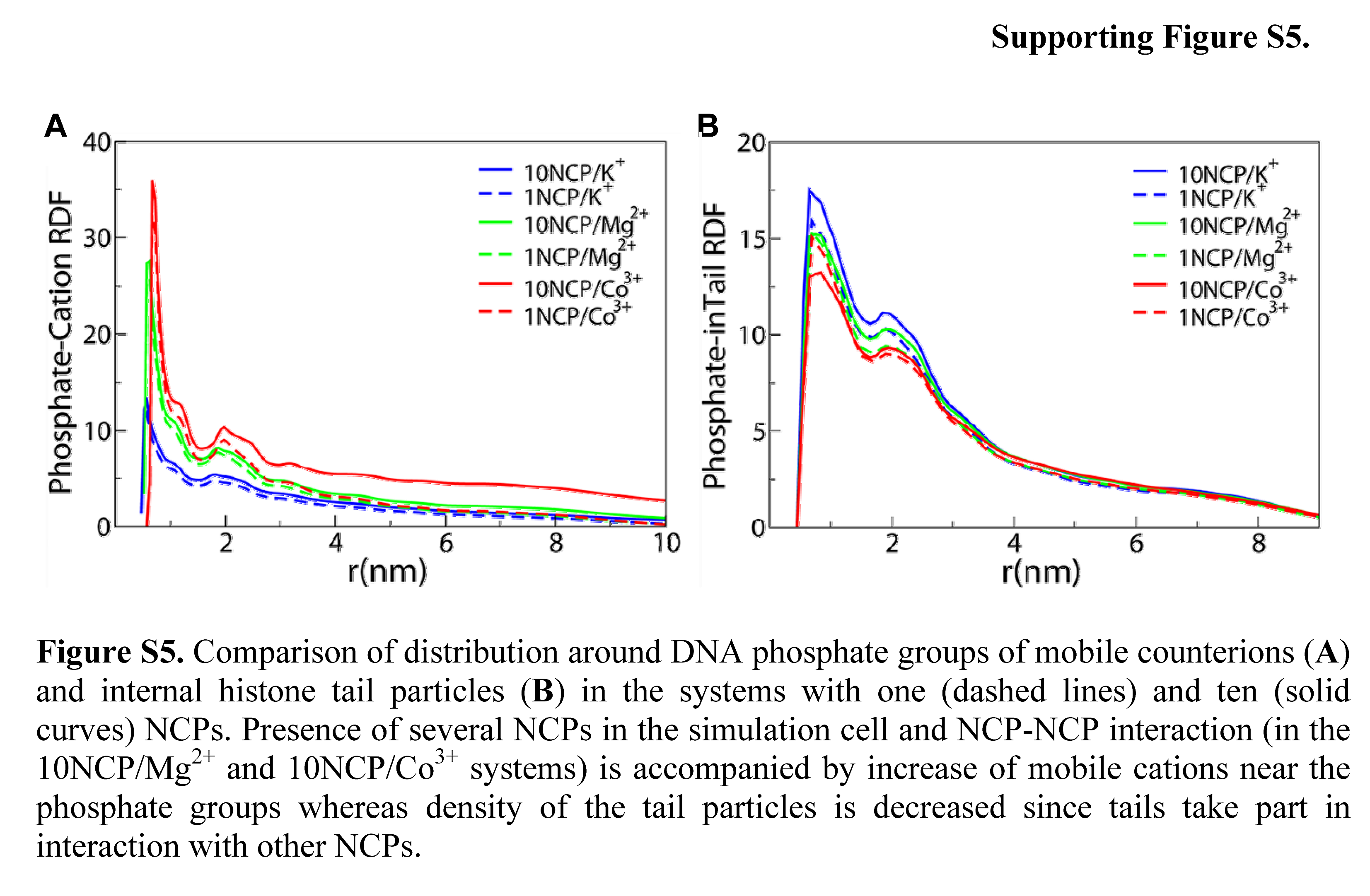

Supplement: Figure S5 — Comparison of mobile counterion distributions around DNA phosphate groups (A) and internal histone tail particles (B) in systems with one and ten NCPs. (TIF) [file pone.0054228.s005.tif]

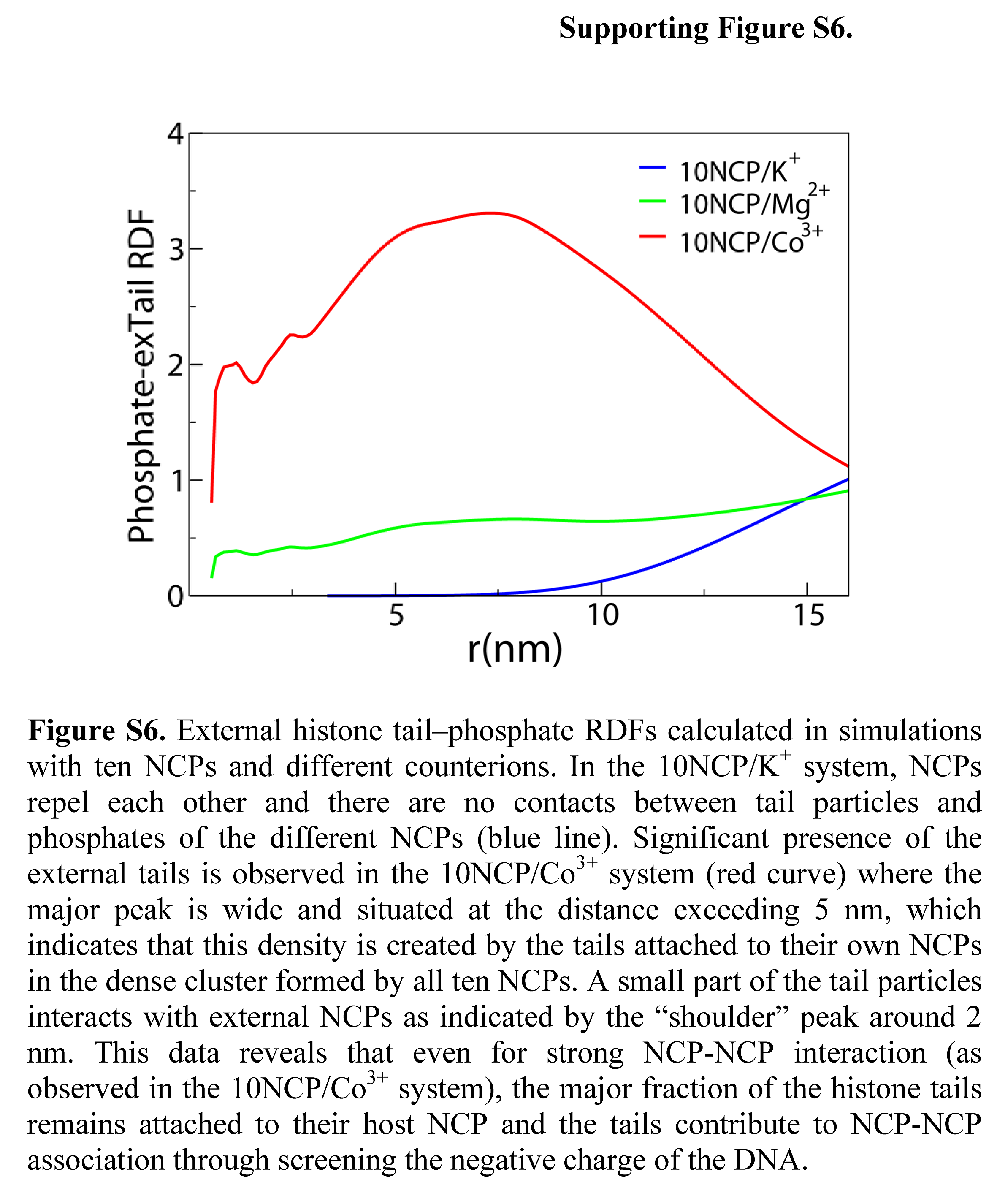

Supplement: Figure S6 — External histone tail–phosphate RDFs calculated in simulations with ten NCPs and different counterions. (TIF) [file pone.0054228.s006.tif]

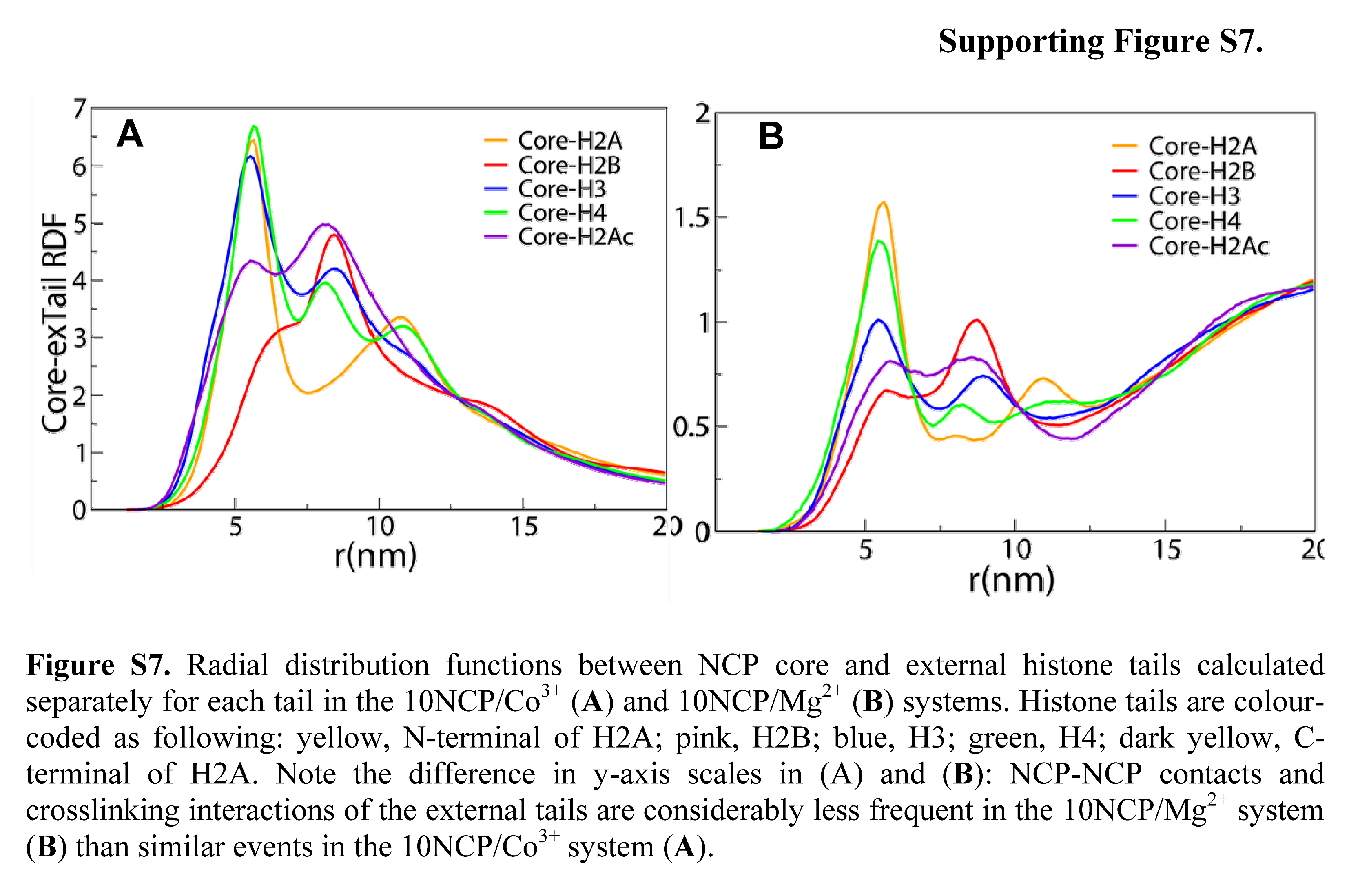

Supplement: Figure S7 — Radial distribution functions between the NCP core and external histone tails calculated separately for each tail in the 10NCP/Co3+ (A) and 10NCP/Mg2+ (B) systems. (TIF) [file pone.0054228.s007.tif]

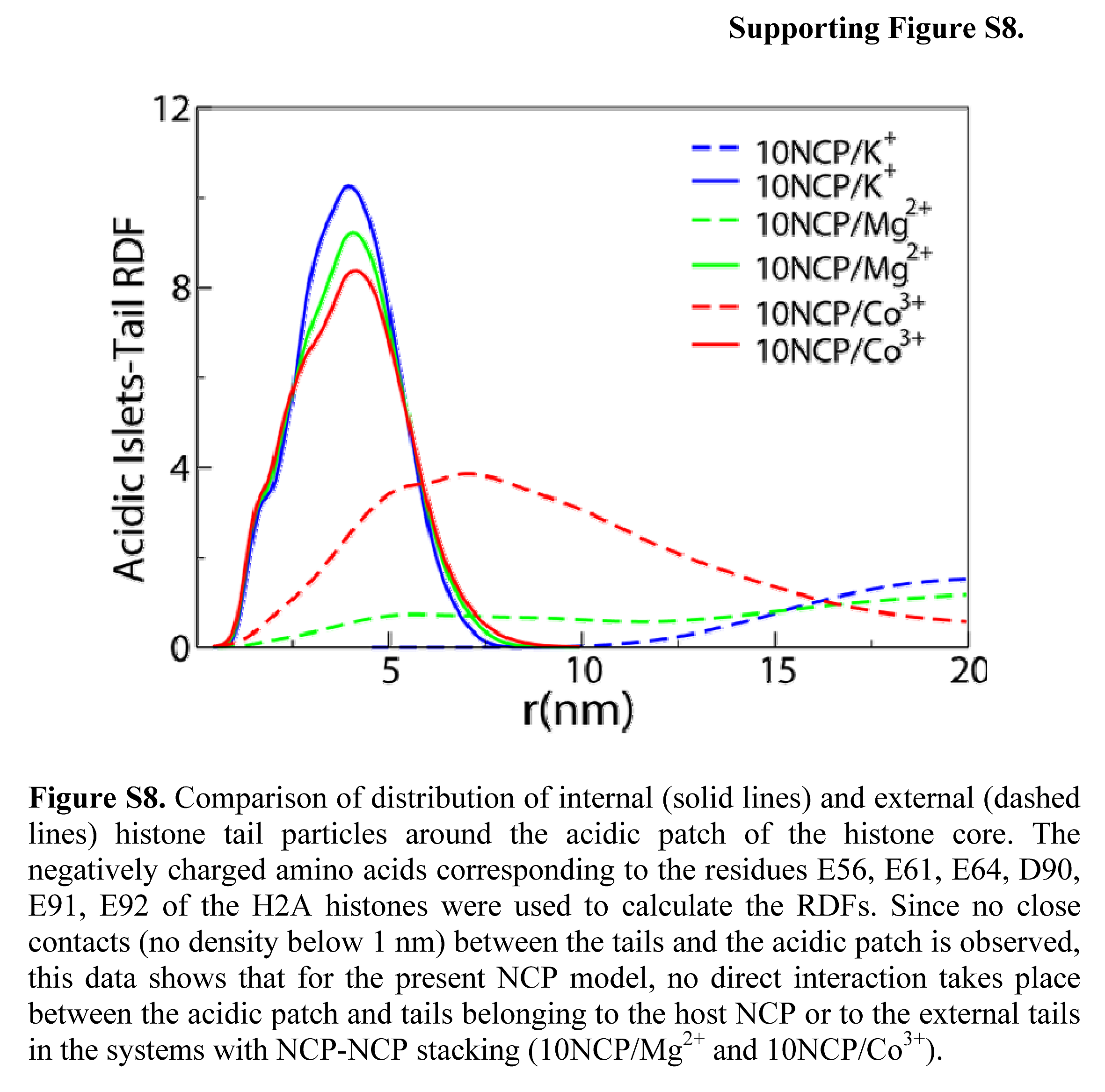

Supplement: Figure S8 — Comparison of distribution of internal and external histone tail particles around the acidic patch of the histone core. (TIF) [file pone.0054228.s008.tif]

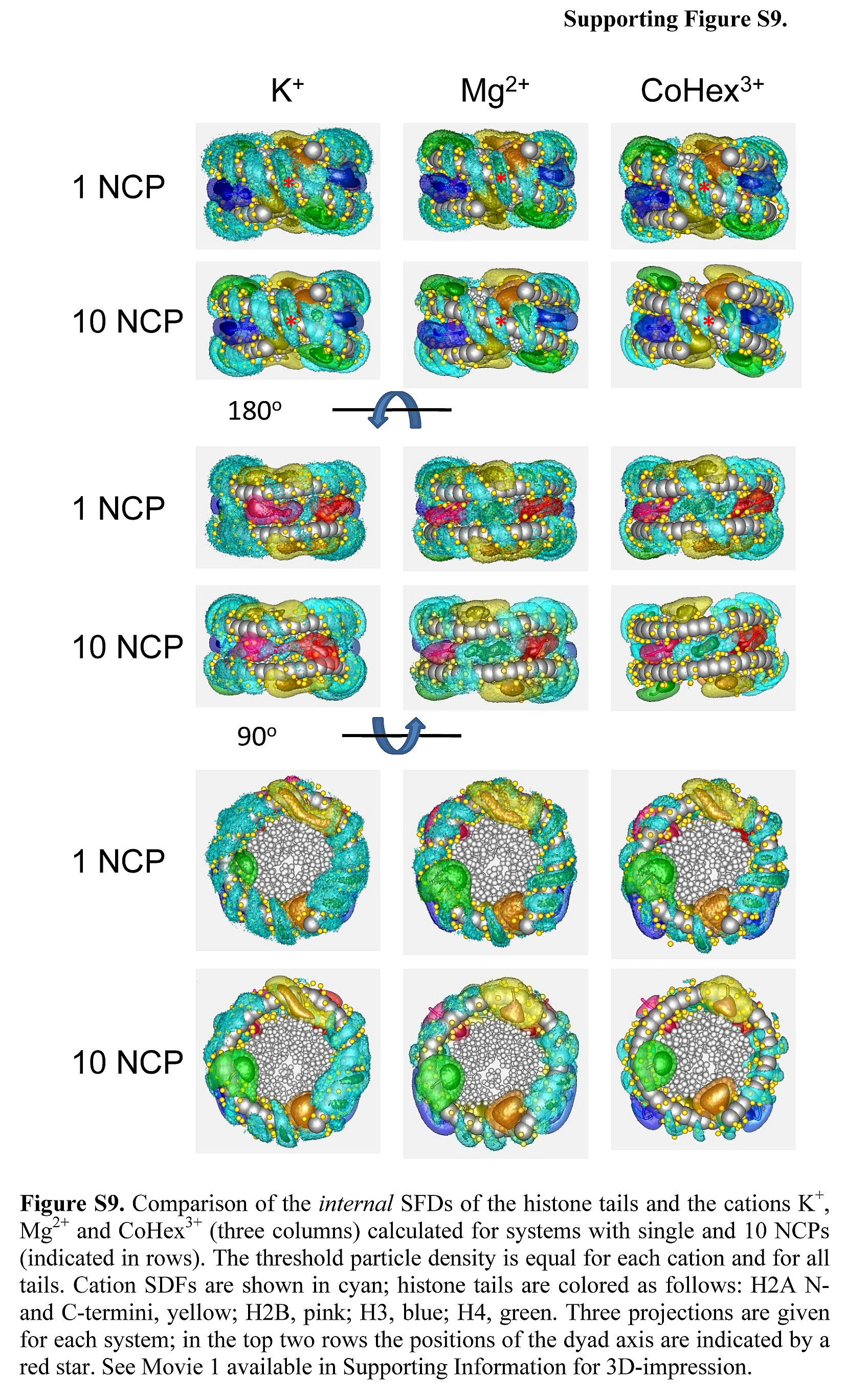

Supplement: Figure S9 — Comparison of the internal SFDs of the histone tails and the cations K+, Mg2+ and CoHex3+ calculated for systems with single and 10 NCPs. (TIF) [file pone.0054228.s009.tif]

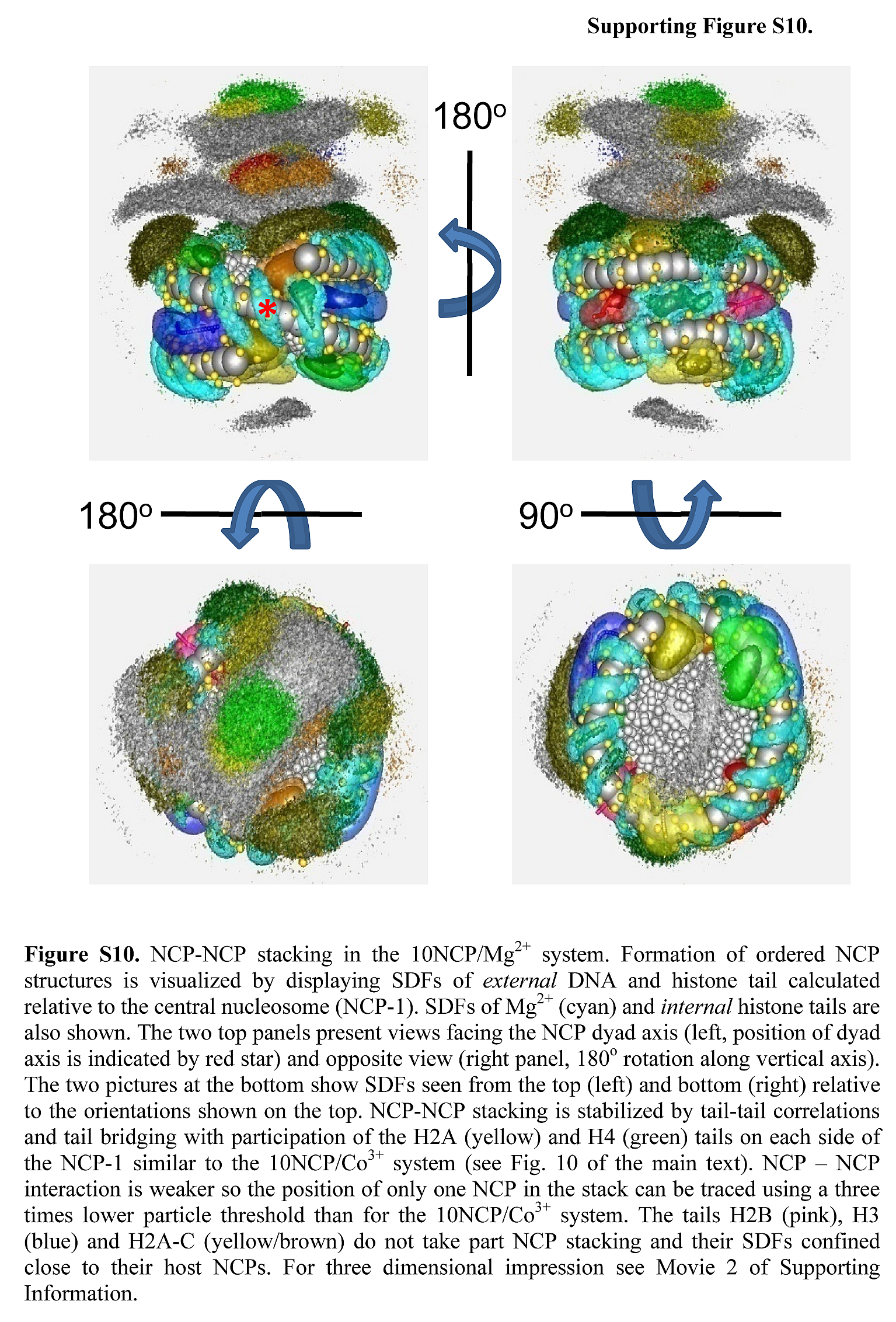

Supplement: Figure S10 — NCP-NCP stacking in the 10NCP/Mg2+ system. Formation of ordered NCP structures is visualized by displaying SDFs of external DNA and histone tails calculated relative to the central nucleosome. (TIF) [file pone.0054228.s010.tif]
